# Serial five-membered lactone ring ions in the treatment of Alzheimer’s diseases-comprehensive profiling of arctigenin metabolites and network analysis

**DOI:** 10.3389/fphar.2022.1065654

**Published:** 2022-12-20

**Authors:** Yanan Li, Xianming Lan, Shaoping Wang, Yifang Cui, Shuyi Song, Hongyan Zhou, Qiyan Li, Long Dai, Jiayu Zhang

**Affiliations:** ^1^ School of Pharmacy, Binzhou Medical University, Yantai, China; ^2^ School of Pharmacy, Shandong University of Traditional Chinese Medicine, Jinan, China; ^3^ Shandong Provincial Institute for Food and Drug Control, Jinan, China

**Keywords:** arctigenin, five-membered lactone ring ions fishing strategy, metabolism, Alzheimer’s diseases, network pharmacology, UHPLC-Q-exactive orbitrap mass spectrometer

## Abstract

Arctigenin is a phenylpropanoid dibenzylbutyro lactone lignan compound with multiple biological functions. Previous studies have shown that arctigenin have neuroprotective effects in Alzheimer’s disease (AD) models both *in vivo* and *in vitro*; however, its metabolism *in vivo* has not been studied. Most traditional analytical methods only partially characterize drug metabolite prototypes, so there is an urgent need for a research strategy that can fully characterize drug metabolites. In the present study, ions fishing with a serial five-membered lactone ring as a fishhook strategy based on ultrahigh-performance liquid chromatography-Q-Exactive Orbitrap mass spectrometry (UHPLC-Q-Exactive Orbitrap MS) was utilised to characterise the metabolism of arctigenin, and the establishment of this strategy also solved the challenge of creating a comprehensive metabolic profile of neolignan. Based on the proposed strategy, a total of 105 metabolites were detected and characterised, 76 metabolites of which were found in rats and 49 metabolites in liver microsomes. These metabolites were postulated to be produced through oxidation, reduction, hydrolysis, and complex reactions. Subsequently, network pharmacology was utilized to elucidate the mechanism of arctigenin and its main metabolites against Alzheimer’s disease, screening 381 potential targets and 20 major signaling pathways. The study on the comprehensive metabolism of arctigenin provides a holistic metabolic profile, which will help to better understand the mechanism of arctigenin in the treatment of Alzheimer’s disease (AD) and also provide a basis for the safe administration of arctigenin.

## Introduction

Arctigenin, 2-(3′-methoxy-4′-hydroxybenzyl)-3-(3″,4″-dimethoxybenzyl) -butyrolactone, can be attributed to natural lignolide compounds. It exists in many botanical drugs and plant-based foods, such as *Arctium lappa* L. (Gao et al., 2018), *Torreya nucifera* L.) Siebold and Zucc. (Yao et al., 2011), *Saussurea medusa* Maxim. (Cho et al., 2004), *Trachelospermum jasminoides* (Lindl.) Lem. (Zhao et al., 2017), *Wikstroemia indica* L.) C. A. Mey. (Kato et al., 2014), and *Forsythia suspensa* (Thunb.) Vahl (Okubo et al., 2020), *etc.* Previous studies have revealed that arctigenin is neuroprotective in AD models both *in vivo* and *in vitro*. In addition, it has significant anti-tumour (Shabgah et al., 2021), anti-inflammatory (Gao et al., 2018), antiviral (Shen et al., 2020), and phytoestrogenic (Hsieh et al., 2014) activities. The mechanism of arctigenin as a potential neuroprotective agent has been elucidated. The pathogenesis of AD is mainly caused by β-amyloid (Aβ)-induced neurodegeneration, and arctigenin can inhibit Aβ by suppressing the expression of β-site amyloid precursor protein cleavage enzyme 1, which in turn delays the attack of Alzheimer’s disease (Jang et al., 2001; Ma et al., 2010). Although arctigenin has been proved effective in treating AD, the metabolism profile has been still unclear, and further studies are needed to determine whether arctigenin metabolites exert potential pharmacological activity in the anti-AD process.

Drug metabolism is the process in which the chemical structure of a drug is changed by the action of various drug metabolism enzymes (especially hepatic drug enzymes) in the body. There are four main pharmacological effects of drug metabolism (Chen et al., 2019): 1) Transforming non-pharmacological substances into active metabolites; 2) Conversion to inactive substances; 3) Changes in pharmacological types of drugs; 4) Production of toxic metabolites. Moreover, as is known to us, the activities of drugs are usually reflected not only in prototype drugs, but also in their metabolites in fact. And thus, the comprehensive metabolism study will help better understand the pharmacological mechanism of arctigenin for AD treatment.

However, there is still no suitable analysis strategy to mine the arctigenin metabolites. Although some databases can provide predictive components, the reliability of predictive values is low and the number of identified metabolites was limited. And thus, the metabolism of arctigenin *in vivo* was partially revealed in the previous studies. Gao et al. identified three arctigenin metabolites, arctigenin acid, arctigenin-4′-O-glucuronide, and 4-O-demethylarctigenin, in rats using LC-MS/MS. (Gao et al., 2013); Jin et al. isolated seven metabolites by incubating arctigenin with *Eubacterium E.) limosum* ARC-2 (Jin et al., 2013); Liu et al. used human intestinal bacteria of AUH-JLD56 to convert arctigenin to 3′-DMAG (3′-Desmethylarctigenin) (Liu et al., 2013). Nevertheless, there was still little information available about arctigenin metabolites in plasma, faeces, urine, and liver microsomes in the above mentioned studies.

Herein, we first proposed a strategy based on Serial Five-Membered Lactone Ring Ions Fishing to find more arctigenin metabolites. We adopted ultrahigh-performance liquid chromatography-Q-Exactive Orbitrap mass spectrometry (UHPLC-Q-Exactive Orbitrap MS) to quickly screen and characterize arctigenin metabolites *in vivo* and *in vitro*. Meanwhile, the ions fishing method based on 5-membered lactone ring or related fragment ions as fishhooks was combined with various data processing tools to establish a comprehensive analytical strategy for characterizing arctigenin metabolites. Moreover, the anti-AD mechanism of main arctigenin metabolites was elucidated by network pharmacology.

## Materials and method

### Chemicals and reagents

Arctigenin reference (Batch number: MUST-21062310, purity ≥99.28%) was provided by Chengdu Must-Technology Co., Ltd (Sichuan, China). HPLC grade acetonitrile, methanol and formic acid (FA) were purchased from Thermo Fisher Scientific (Fair Lawn, NJ, United States), and deionized water for analysis was purchased from Watson (Jinan, China). N_2_ required for the instrument is available at the workstation of the Shandong Institute of Traditional Chinese Medicine (Jinan, China).

Pooled male Sprague Dawley (SD) rat liver microsomes (1 ml, Batch number: 20,210,305), uridine diphosphoglucuronic acid trisodium salt (UDPGA), nicotinamine adenine dinucleotide phosphate (NADPH), and MgCl_2_ were all purchased from NEWGAINBIO Co., Ltd (Wuxi, China). Oasis^®^ HLB C_18_-Low solid-phase extraction cartridges (500 mg, 6 ml^−1^, 60 μm, 149 Å) were purchased from Waters Corporation (Milford, USA). 6-Well plates were obtained from Corning Incorporated-Life Science (Jiangsu, China).

### Animals and drug administration

Eight male SD rats (200 ± 10 g) were supplied by Jinan Pengyue Experimental Animal Company (Jinan, China). They were free to eat and drink at a constant temperature of 25 ± 2°C and a humidity of 55 ± 10% for 7 days. Eight rats were randomly divided into two groups: Drug Group (*n* = 4) for test plasma, urine, faeces, and liver; Control Group (*n* = 4) for blank plasma, urine, faeces, and liver. The animal protocols were approved by the institutional Animal Care and Use Committee at BIN ZHOU Medical University (2021-085). The animal facilities and protocols were complied with the Guide for the Care and Use of Laboratory Animals (USA National Research Council, 1996). The above experimental conditions were in agreement with our previous report (Jiang et al., 2021).

### *In Vivo* animal experiment

Arctigenin was dissolved in 0.9% saline solution and administered to rats in the Drug Group (150 mg kg^−1^, 10 ml kg^−1^), compared to Control Group rats that were given equivalent volume of 0.9% saline solution. Blood samples (0.5 ml) were taken from the suborbital venous plexus at 0.5, 1, 2, 4, and 6 h after arctigenin administration. The plasma samples were prepared by centrifuging at 3,500 rpm for 10 min. Additionally, the urine and faeces samples were collected in a metabolic cage within 24 h after administration. Finally, the rats were dissected and liver samples were gathered. All homogeneous biological samples from the same group were merged into a collective sample (Dong F. et al., 2021).

### Sample pretreatment

The SPE cartridges for plasma, urine and faeces samples were the same as the previous treatments (Dong P. et al., 2021; Xiang et al., 2021; Jing et al., 2022). In addition, plasma samples were processed by two other methods. Methanol: Plasma samples (1 ml) were added with methanol (3 ml) and centrifuged for 5 min (14,000 rpm, 4°C) to take the supernatant. Acetonitrile: Plasma samples (1 ml) were added with acetonitrile (3 ml) and centrifuged for 5 min (14,000 rpm, 4°C) to obtain the supernatant. Furthermore, liver samples (1 g) were added with physiological saline (10 ml) for grinding and centrifuged for 5 min (3,500 rpm, 4°C) to obtain the supernatant, and SPE cartridges was used for liver samples preparation. Finally, all samples were dried under N_2_ at room temperature.

### *In Vitro* liver microsomes incubation

Firstly, the incubation mixture was prepared in PBS buffer containing rat liver microsomes (1 mg mL^−1^), arctigenin (0.1 mg mL^−1^), and MgCl_2_ (3 mM). To set up the Drug Group and the Control Group, 900 μL of the incubation mixture was added to each hole of the 6-well plate, while the Control Group was given the negative drug solution. After incubation for 5 min at 37°C, 100 μL NADPH (25 mg mL^−1^) and 100 μL UDPGA (25 mg mL^−1^) were added to the incubation mixture to start the phase I and II reaction. Then continue to incubate at 37°C, 200 μL cold acetonitrile was added to 100 μL the incubation mixture to terminate the reaction at 5, 10, 15, 30, 45, 60, 120, 240 min, respectively. Finally, the supernatant was centrifuged, and the subsequent experiment were performed in the same method as the acetonitrile precipitation of plasma samples.

### Instruments and analytical conditions

The chromatographic separation was performed on DIONEX Ultimate 3000 UHPLC system (Thermo Fisher Scientific, MA, United States). Separation was performed on a Waters ACQUITY UPLC BEH C18 column (2.1 × 100 mm, 1.7 μm). The column temperature was kept at 35°C and the injection volume was 3 μL. The mobile phase was made up of 0.1% formic acid A) and acetonitrile B). The elution gradient was set as follows: 0–5 min, 5%–30% B; 5–10 min, 30%–50% B; 10–27 min, 50%–90% B; 27–27.1 min, 90%–5% B; 27.1–30 min, 5% B.

HRMS and MS/MS spectra were acquired using Q-Exactive Focus Orbitrap MS (Thermo Fisher, Waltham, MA, USA) equipped with a heated electrospray ionization source. All samples were analyzed in positive and in negative ion modes. The ion source parameters were set as follows: spray voltage, 3.8/3.5 kV (+/-); sheath gas flow rate, 45 arbitrary units; Aux gas flow rate, 10 arbitrary units; capillary temperature, 320°C; Aux gas heater temperature, 320°C; scan modes, full MS resolution, 70,000; dd-MS^2^ resolution, 17,500; scan range, *m/z* 80-1,200.

### Peak selections and data processing

The Thermo Xcalibur 2.1 workstation was used to process the collected data set. The chemical formulae and exact masses of the parent ions of the selected peaks were predicted by setting the following parameters: C [0-40], H [0-50], O [0-20], S [0-4], N [0-4] and ring double bond (RDB) equivalent values [0-15]. The mass error range was set to within ±5 ppm.

### Mechanism study on anti-AD about metabolites based on network pharmacology

#### Prediction of arctigenin targets for AD treatment

The arctigenin metabolites currently reported in the literatures were compiled (Hao et al., 2013), their SMILE was searched, and imported into the Swiss target prediction database (Daina et al., 2019). Then, the targets related to AD were obtained in the GeneCards database. Finally, the potential therapeutic targets of metabolites for AD were obtained.

#### Construction of the protein-protein interaction networks of the major targets

In order to study the interactions between target proteins of arctigenin metabolites for treating AD, the main targets were imported into String Version 11.0 platform. A PPI network diagram was constructed using Cytoscape 3.9.1, and core targets were screened based on degree value.

#### Enrichment analysis of the kyoto encyclopedia of genes and genomes pathways and gene ontology pathways

Using the Metascape database, GO and KEGG analyses were performed to screen the key pathways for the main targets of arctigenin metabolites for treating AD.

#### Construction of disease-pathways-targets-metabolites network

The connection between targets, pathways, diseases, and metabolites was not clear, so it was necessary to construct a disease-pathways-targets-metabolites network. Firstly, diseases, major pathways, metabolites, and key targets were imported into Cytoscape 3.9.1 software. Then, the parameters were adjusted and visualized for analysis. Finally, the disease-pathways-targets-metabolites network diagram was constructed.

## Results

### Metabolites identification workflow

**FIGURE 1:**
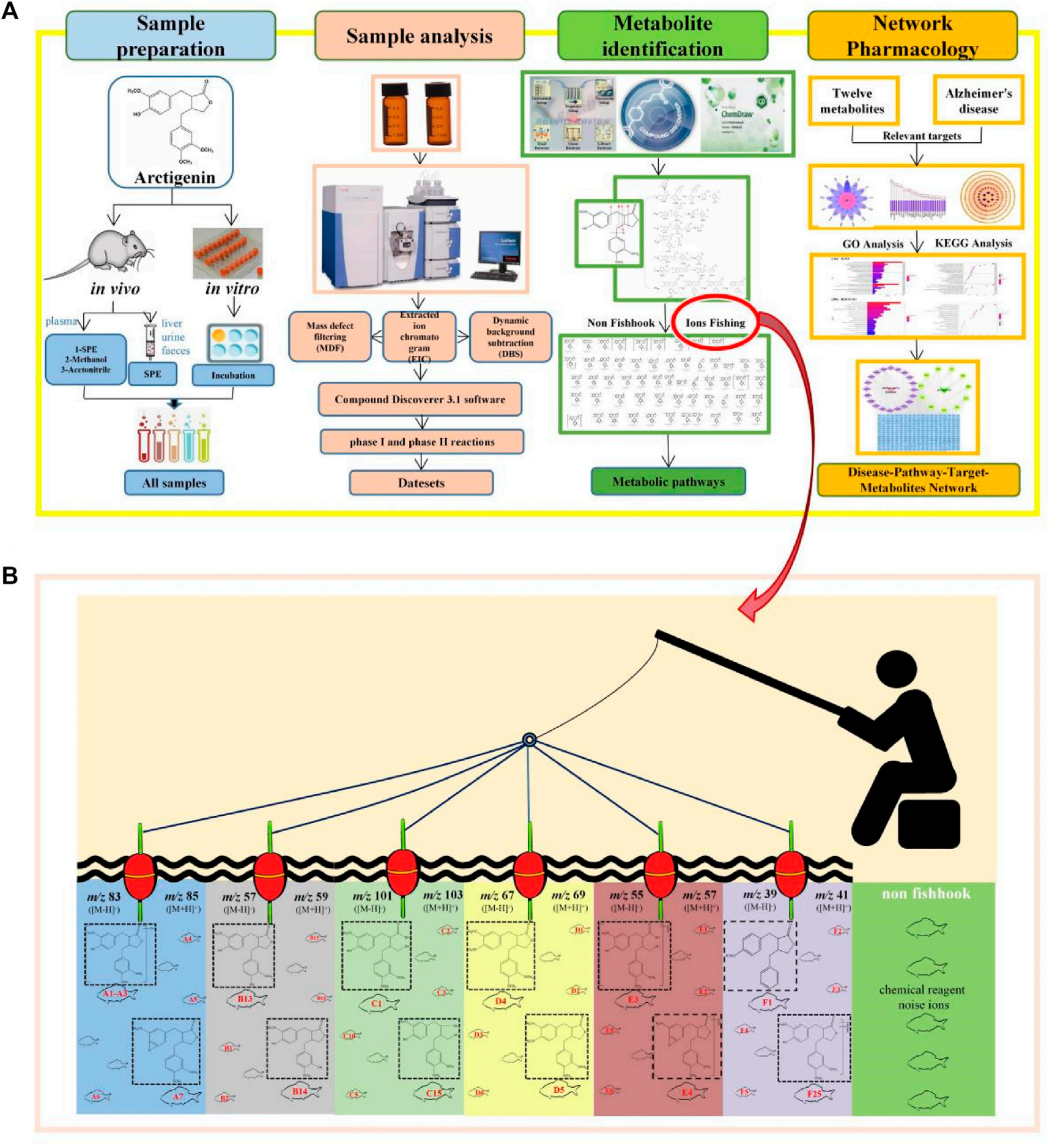
Workflow of the analytical procedure for identification of arctigenin metabolites **(A)** General workflow **(B)** Ions fishing.

The workflow (Figure 1A) was divided into four steps to characterize the arctigenin metabolites and their anti-AD mechanism. First, biological samples were prepared through *in vivo* and *in vitro* metabolism coupled with different sample processing methods. Second, after the full-scan data was acquired online, all the potential metabolites data was obtained. ESI-MS/MS spectra of the control samples and the drug samples were imported into the Compound Discoverer 3.1 workstation in turn to establish metabolites fishpond. The components (or ions) named “troublemakers” from the control samples would be eliminated in the drug samples. Multiple data processing methods, such as Generate Expected Compound, Create FISh Trace, Search mzVault, and Compound Class Scoring, in Compound Discoverer 3.1 software were adopted to filter the possible arctigenin metabolites. It was worth mentioning that all types of phase I and phase II reactions could be selected in the Generate Expected Compound parameter setting, which was conducive to classifying arctigenin metabolites. The third step was metabolites identification. Using Thermo Xcalibur 2.1, Compound Discoverer 3.1, Chemraw 14.0, and other software, the identification process of metabolites was described based on their molecular mass, cleavage pathways of arctigenin and biotransformation information. Then, the arctigenin metabolites were screened and identified by the ion fishing strategy. Finally, a network pharmacological analysis of arctigenin metabolites reported in the literatures was performed to establish the disease-pathways-targets-metabolites network diagram. Thereby, the mechanism of arctigenin metabolites in the treatment of AD was elucidated.

### The establishment of serial five-membered lactone ring ions fishing-based analytical strategy

**FIGURE 2:**
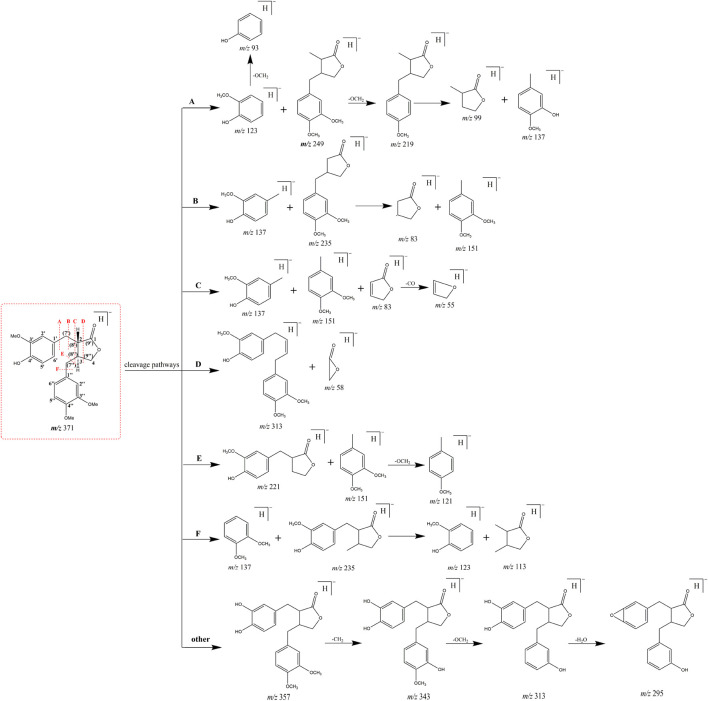
Typical cleavage pathways for arctigenin.

Arctigenin, a compound based on the backbone of 2, 3-dibenzyl-butyrolactone, consists of three methoxy groups and one hydroxy group. Combined with its structural features, debenzylation and the neutral loss of methyl and methoxy groups should be the major fragmentation pathways. More importantly, we analysed, compared and summarized the characteristic mass fragmentation behaviour of arctigenin (Figure 2) to obtain the elimination patterns. It was found that each cleavage pathway could produce five-membered lactone ring or its derived fragment ions, which were *m/z* 83 [M-H]^-^/*m/z* 85 [M + H]^+^ (five-membered lactone ring), *m/z* 57 [M-H]^-^/*m/z* 59 [M + H]^+^ (*m/z* 83-C_2_H_4_/*m/z* 85-C_2_H_4_), *m/z* 101 [M-H]^-^/*m/z* 103 [M + H]^+^ (*m/z* 83-H_2_O/*m/z* 85-H_2_O), *m/z* 55 [M-H]^-^/*m/z* 57 [M + H]^+^ (*m/z* 83-CO/*m/z* 85-CO), *m/z* 67 [M-H]^-^/*m/z* 69 [M + H]^+^ (*m/z* 83-O/*m/z* 85-O), and *m/z* 39 [M-H]^-^/*m/z* 41 [M + H]^+^ (*m/z* 83-CO_2_/*m/z* 85-CO_2_). Based on this, we proposed the concept of “ions fishing” with 5-membered lactone ring and related fragments as fishhooks to screen the metabolites of arctigenin. In other words, these fragment ions were regarded as fishhooks to accurately “fish” the arctigenin metabolites in the fishpond of the retrieved metabolites by Compound Discoverer 3.1 software. In addition, some metabolites of the de-lactone ring were found in the fishpond.

The acquired data were processed using the above strategy and most of the interfering ions were found to be significantly reduced. The fact that 105 metabolites were identified in this study demonstrated the feasibility of the strategy, and also provided a basis for the identification of neolignan metabolites.

### Mass cleavage pattern of arctigenin

**FIGURE 3:**
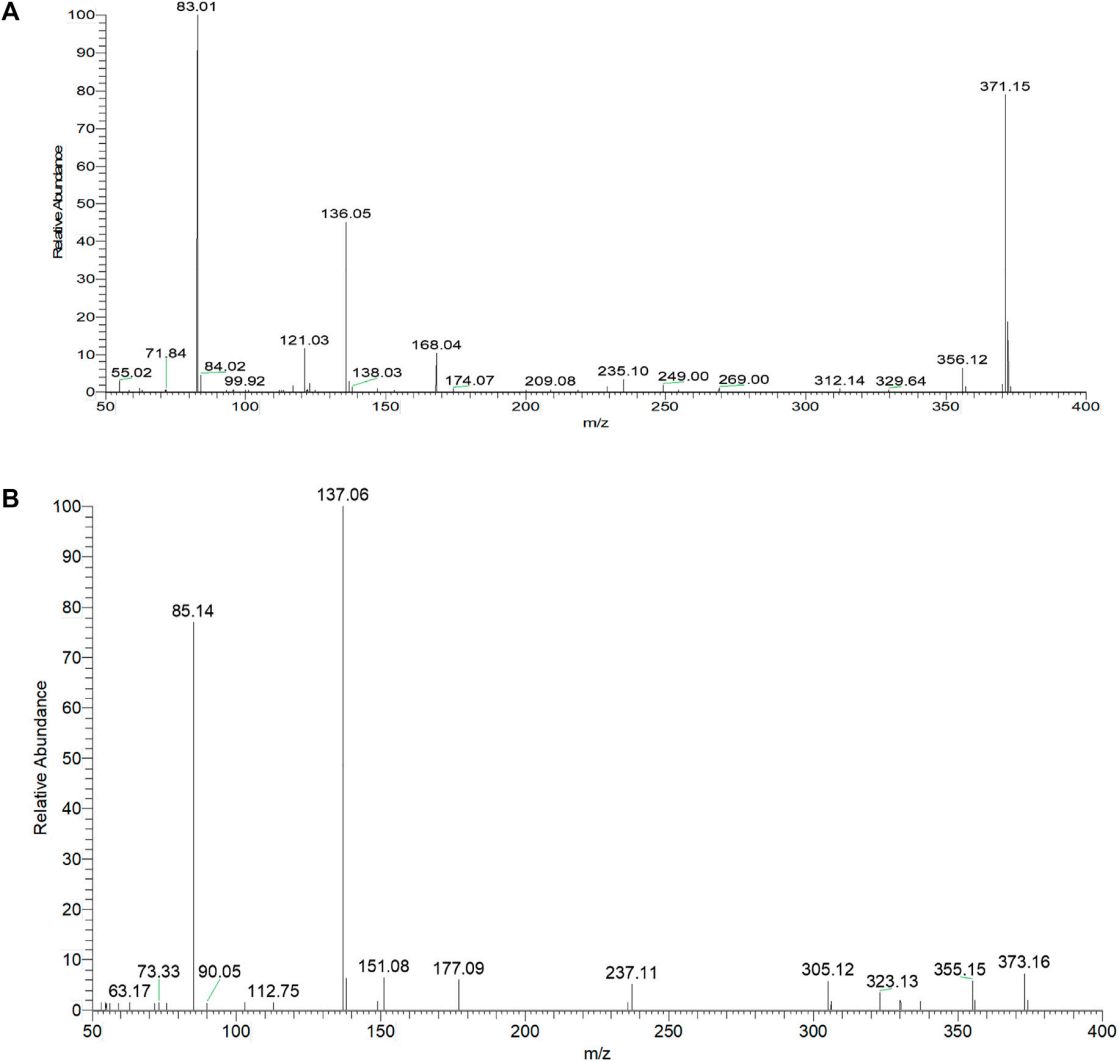
The ESI-MS^2^ spectra of arctigenin in **(A)** negative and **(B)** positive ion modes.

Based on the retention time at 9.83 min, arctigenin generated [M + H]^+^ and [M-H]^-^ ions at *m/z* 373.16364 and *m/z* 371.15009, respectively. To guide the subsequent rapid analysis, the parent drug (arctigenin) with the chemical formula C_21_H_23_O_6_ (-, mass error of 0.774 ppm) and C_21_H_25_O_6_ (+, mass error of -0.479 ppm) was unambiguously identified by analyzing the splitting behavior of arctigenin standard. In the ESI-MS^2^ spectra (Figure 3), arctigenin mainly occurred benzyl cleavage; that is, the C-C bond between C7’ (C7″) and C8’ (C8″) was broken. In addition, arctigenin also owned a variety of cleavage modes, and several main cleavage pathways were marked in Figure 2. For example, in negative ion mode, cleavage pathway A): Two characteristic ions at *m/z* 123 and *m/z* 249 were observed after the cleavage of C6′-C7′ bond. Additionally, two dominant fragment ions at *m/z* 83 and *m/z* 235 were generated after successive losing of OCH_2_ and CH_2_. Cleavage pathway B): Three characteristic ions of *m/z* 137, m*/z* 83, and *m/z* 152 were generated by debenzylation of the C7′-C8′ bond. Cleavage pathway C): Debenzylation, which mainly broke the C7′-C8′ bond and C7″-C8″ bond to form the characteristic ion at *m/z* 83, which was unstable and then formed fragment ions at *m/z* 67 (83-O) or *m/z* 55 (83-CO) or *m/z* 39 (83-CO_2_). Cleavage pathway D): The *m/z* 57 was present in the ESI-MS^2^ spectra. Cleavage pathway E): The breaking of C7″-C8″ bond produced characteristic ions at *m/z* 221 and *m/z* 122. Cleavage pathway F): Several ions at *m/z* 137, m*/z* 123, and *m/z* 113 were present in the ESI-MS^2^ spectra. Other pathways: In addition to the above cracking methods, in negative ion mode, a series of DPIs could be yielded by losing CH_2_, CO, OH, CH_3_, H_2_O, and their combinations, such as *m/z* 357 [M-H-CH_2_]^-^, *m/z* 329 [M-H-CH_2_-CO]^-^, *m/z* 312 [M-H-CH_2_-CO-OH]^-^, *m/z* 269 [M-H-2CH_2_-2CO-H_2_O]^-^, *m/z* 254 [M-H-2CH_2_-2CO-H_2_O-CH_3_]^-^. The fragment ions of serial five-membered lactone ring produced various cleavage pathways of arctigenin could be used as vital fishhooks to hook arctigenin metabolites.

### Summary of arctigenin metabolites

**FIGURE 4:**
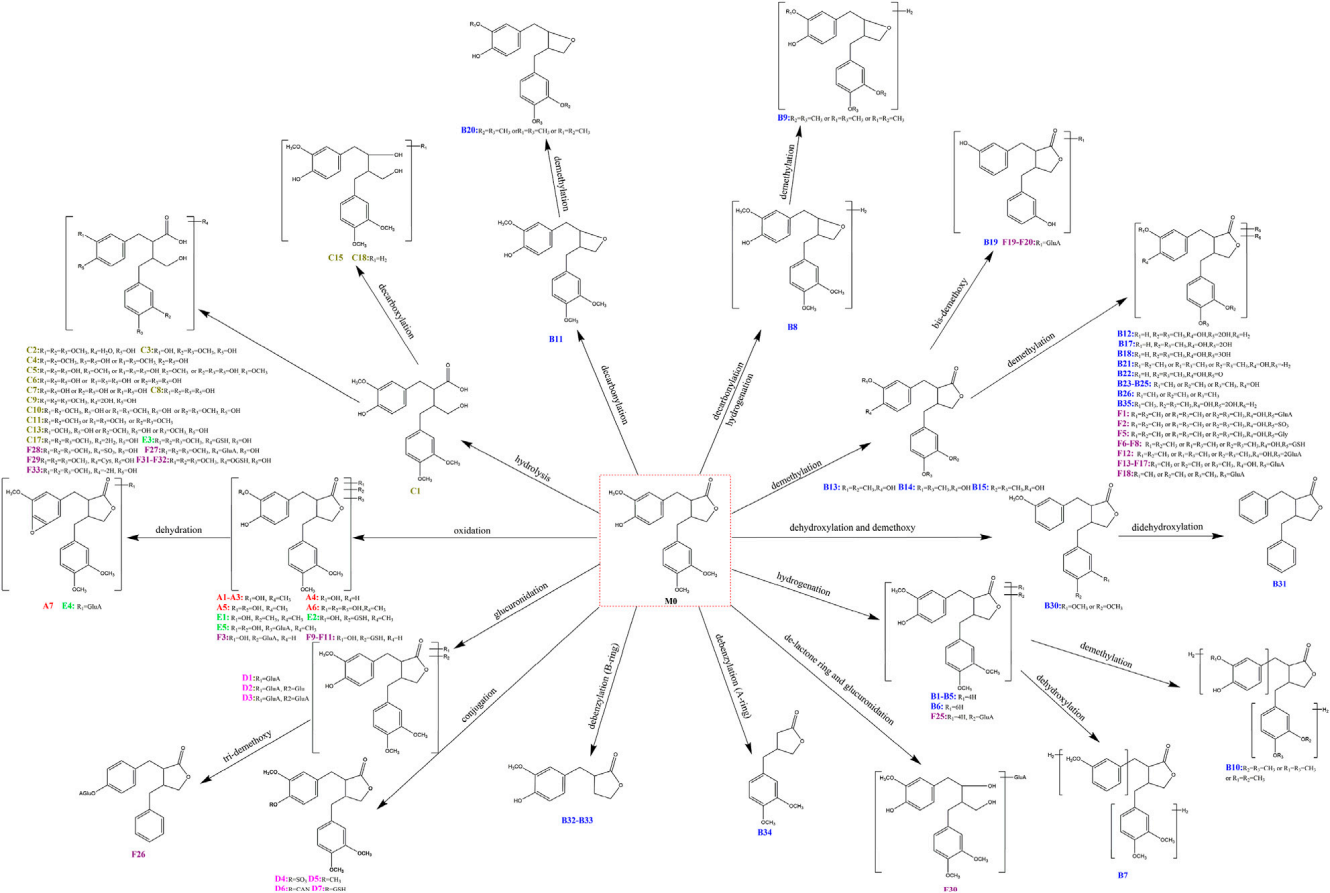
The proposed metabolic pathways of 105 identified arctigenin metabolites.

As a result, based on five-membered lactone ring ions fishing analytical strategy, 105 metabolites were identified in rat plasma, urine, faeces, liver and liver microsomes samples. According to the classification of ions fishing, Supplementary Table S1 provided all fished arctigenin metabolites. All structures of metabolites were shown in Figure 4.

Representative Metabolites of Fishhook with *m/z* 83/*m/z* 85 ([M-H]^-^/[M + H]^+^)

**A1**, **A2**, and **A3** possessed the same theoretical [M-H]^-^/[M + H]^+^ ions at *m/z* 387.14383 (C_21_H_23_O_7_, mass errors within ±5 ppm)/*m/z* 389.15947 (C_21_H_25_O_7_, mass errors within ±5 ppm) in negative and positive ion modes, which were eluted at 6.70, 8.38, and 8.45 min, respectively. They were 16 Da more massive than arctigenin, indicating that they might be mono-oxidised products of arctigenin. In their ESI-MS^2^ spectra, a fishing ion of *m/z* 83 was observed through the cleavage pathway C). In addition, several dominant fragment ions at *m/z* 124, m*/z* 137, and *m/z* 387 were observed through the cleavage pathway F). Two characteristic ions at *m/z* 235 and *m/z* 250 were observed in the ESI**-**MS^2^ spectra, suggesting that **A1** was A-ring mono-oxidised metabolite of arctigenin. Moreover, some typical ions at *m/z* 151 and *m/z* 167 appeared in the ESI-MS^2^ spectra of **A2** and **A3**, confirming that the B ring contained the added O atom.

With the retention time of 7.08 min, **A4** displayed the deprotonated molecular ion at *m/z* 373.12985 (C_20_H_21_O_7_, mass error of 1.538 ppm) in negative ion mode, and it was 14 Da less massive than **A1**-**A3**, which suggested that demethylation and oxidation reaction were present. It showed fragment ions at *m/z* 329 [M-H-CO_2_]^-^, *m/z* 299 [M-H-CO_2_-CH_2_-2OCH_2_]^-^, *m/z* 284 [M-H-CO_2_-CH_2_-CH_3_-2OCH_2_]^-^, *m/z* 150 [M-H-C_11_H_11_O_4_^−^-O]^-^, *m/z* 136 [M-H-C_11_H_11_O_4_-O-CH_2_]^-^, *m/z* 121 [M-H-C_11_H_11_O_4_-O-CH_2_-CH_3_]^-^, and *m/z* 109 [M-H-C_14_H_16_O_5_]^-^. Among them, *m/z* 121 and *m/z* 109 showed that demethylation reaction occurred on A-ring, and hydroxylation reaction occurred on B-ring. Therefore, **A4** was deduced as the demethylation and oxidation metabolite of arctigenin.

**A5** gave rise to the deprotonated ion at *m/z* 403.13943 (C_21_H_23_O_8_, mass error of -0.019 ppm) with retention time of 10.50 min in negative ion mode. It was 32 Da more massive than arctigenin. Most importantly, product ion at *m/z* 83 was fished in the ESI-MS^2^ spectrum of **A5**. Moreover, some representative ions at *m/z* 233 [M-H-C_8_H_8_O_3_-H_2_O]^-^, *m/z* 151 [M-H-C_13_H_16_O_5_]^-^, *m/z* 139 [M-H-C_14_H_16_O_5_]^-^, *m/z* 137 [M-H-C_13_H_16_O_5_-CH_2_]^-^, and 123 [M-H-C_14_H_16_O_5_-O]^-^ appeared in the ESI-MS^2^ spectrum, which indicated that **A5** was A-ring and B-ring di-oxidation metabolite of arctigenin.

**A6** (*m/z* 419.13516, C_21_H_23_O_9_, mass error of 0.965 ppm) was eluted at 6.96 min in negative ion mode. It was 48 Da more massive than arctigenin, implying tri-hydroxylation reaction might occur. It produced fragment ions at *m/z* 352 [M-H-OCH_3_-2H_2_O]^-^, *m/z* 252 [M-H-C_9_H_11_O_3_]^-^, *m/z* 243 [M-H-C_7_H_8_O_3_-2H_2_O]^-^, and *m/z* 123 [M-H-C_14_H_16_O_6_-O]^-^, indicating that hydroxylation reaction occurred on the A-ring and di-hydroxylations reaction occurred on the B-ring. The classical ion at *m/z* 83 was produced by the cleavage pathway C). So **A6** could be tentatively deduced as tri-hydroxylation product of arctigenin.

**A7** (*m/z* 369.13470, C_21_H_21_O_6_, mass error of 0.917 ppm) was 18 Da less massive than **A1**-**A3** with retention time of 9.65 min in negative ion mode. It showed pivotal fragment ions at *m/z* 354 [M-H-CH_3_]^-^, *m/z* 235 [M-H-C_8_H_6_O_2_]^-^, *m/z* 218 [M-H-C_8_H_6_O_2_-OH]^-^, and *m/z* 203.03 [M-H-CH_3_-C_8_H_6_O_2_-OH]^-^. Besides, the cleavage pathway C) produced three typical fragment ions at *m/z* 151, m*/z* 136, and *m/z* 83, which confirmed that hydroxylation and dehydration occurred on A-ring, while hydroxylation occurred at 5-position. Hence, **A7** could be temporarily speculated as hydroxylation and dehydration product of arctigenin.

### Representative Metabolites of Fishhook with *m/z* 57/*m/z* 59 ([M-H]^-^/[M + H]^+^)

**B13**, **B14**, and **B15** showed their theoretical deprotonated molecular ions at *m/z* 357.13321 (C_20_H_21_O_6_, mass error within ±5 ppm), which was 14 Da more massive than arctigenin. Thus, they might be deduced as demethylation metabolites of arctigenin. Five predominant fragment ions at *m/z* 235 [M-H-C_7_H_6_O_2_]^-^, *m/z* 221 [M-H-C_8_H_8_O_2_]^-^, *m/z* 136 [M-H-C_12_H_13_O_4_]^-^, *m/z* 121 [M-H-C_13_H_16_O_4_]^-^, and *m/z* 57 [M-H-C_9_H_11_O_2_-C_7_H_7_O_2_-C_2_H_2_]^-^ provided evidence. Therefore, **B13** (Clog*P* value 1.5850) was interpreted as matairesinol (Kasper et al., 1994), **B14** (Clog*P* value 1.5854) was identified as 3″-O-demethyl-arctigenin (Jin et al., 2013), while **B15** (Clog*P* value 1.6150) was characterized as 3′-O-demethyl-arctigenin (Jin et al., 2007).

**B19** yielded significant [M-H]^-^ ion at *m/z* 297.11346 (C_18_H_17_O_4_, mass error of 0.766 ppm), which was 60 Da less massive than **B13**-**B15**, and *m/z* 57 was fished, suggesting it could be demethylation and bis-demethoxy metabolite of arctigenin. The fragment ions at *m/z* 253 [M-H-2OH]^-^, *m/z* 209 [M-H-2OCH_2_-2CH_2_]^-^, *m/z* 134 [M-H-C_9_H_7_O_3_]^-^, *m/z* 121 [M-H-C_10_H_8_O_3_]^-^, and *m/z* 107 [M-H-C_11_H_10_O_3_]^-^ were observed. According to the literature (Jin et al., 2013), **B19** was deduced to be enterolactone.

**B23**, **B24**, and **B25** possessed the same theoretical [M-H]^-^ ion at *m/z* 343.11957 (C_19_H_19_O_6_, mass errors within ±5 ppm), which were 28 Da less massive than arctigenin. The fragment ions at *m/z* 219 [M-H-C_6_H_4_O_2_-O]^-^, *m/z* 149 [M-H-C_10_H_10_O_4_]^-^, *m/z* 137 [M-H-C_11_H_10_O_4_]^-^, *m/z* 121 [M-H-C_12_H_14_O_4_]^-^, and *m/z* 57 [M-H-C_7_H_7_O_2_-C_8_H_9_O_2_-C_2_H_2_]^-^ were detected in their ESI-MS^2^ spectra, and Cleavage pathway B) \ C) \ E) \ F) were involved. Thus, **B23**, **B24**, and **B25** were determined to be isomeric demethylation metabolites of arctigenin.

Two isomeric metabolites, **B27** and **B28** were 14 Da less massive than **B23**-**B25**, which respectively afforded [M-H]^-^ ions at *m/z* 329.10333 and 329.10330. Both of them showed the same elemental composition of C_18_H_17_O_6_ (mass errors within ±5 ppm) according to Compound Discoverer 3.1 software. Three notable ions at *m/z* 311, m*/z* 285, and *m/z* 121 formed by successive loss of H_2_O, CO_2_, and C_11_H_12_O_4_, respectively. Two other product ions at *m/z* 137 [M-H-C_10_H_8_O_4_]^-^ and *m/z* 165 [M-H-C_8_H_8_O_2_-CO]^-^ were also detected in ESI-MS^2^ spectrum of **B28**. These results suggested that **B27** and **B28** were tri-demethylation metabolites of arctigenin. The Clog*P* values of **B27** and **B28** were 0.6930 and 0.7602, respectively. According to the literature (Jin et al., 2007), **B27** and **B28** were respectively deduced as 3′,3″,4″-O-tridemethyl-arctigenin and 4′,4″-dihydroxy-enterolactone.

### Representative Metabolites of Fishhook with *m/z* 101/*m/z* 103 ([M-H]^-^/[M + H]^+^)

Five-membered lactone ring of arctigenin was easily hydrolyzed to produce ion at *m/z* 101/*m/z* 103 ([M-H]^-^/[M + H]^+^), which could be used to fishing arctigenin metabolites. One deprotonated metabolite of **C1** ion at *m/z* 389.16119 presented a molecular composition of C_21_H_25_O_7_ (mass error of 1.577 ppm) and a retention time of 7.43 min in negative ion mode. **C1** was 18 Da more massive than arctigenin. Five characteristic ions at *m/z* 345 [M-H-CO_2_]^-^, *m/z* 330 [M-H-CO_2_-CH_3_]^-^, *m/z* 253 [M-H-C_8_H_8_O_2_]^-^, *m/z* 219 [M-H-C_8_H_8_O_2_-2OH]^-^, and *m/z* 121 [M-H-C_13_H_18_O_5_-CH_2_]^-^ were obtained. Moreover, key fragment ions at *m/z* 195, 135, and 101 were found. Thus, **C1** was deduced as hydrolysis metabolite of arctigenin. According to the literature (Gao et al., 2014), **C1** was identified as arctigenic acid. **C2** produced the theoretical [M-H]^-^ ion at *m/z* 407.17111 (C_21_H_27_O_8_, mass error of -0.076 ppm) with the mass being 18 Da more than **C1**. Therefore, **C2** could be deduced as bis-hydrolysis metabolite of arctigenin.

### Representative Metabolites of Fishhook with *m/z* 67/*m/z* 69 ([M-H]^-^/[M + H]^+^)

After the five-membered lactone ring of arctigenin lost O, fragment ions at *m/z* 67/*m/z* 69 ([M-H]^-^/[M + H]^+^) was generated for fishing arctigenin metabolites. For example, **D1** showed the theoretical [M-H]^-^ ion at *m/z* 547.18286 (C_27_H_31_O_12_, mass error of 1.390 ppm), which was 176 Da more massive than arctigenin, revealing that a glucuronidation reaction occurred. In the ESI-MS^2^ spectrum, the fragment ion at *m/z* 371 [M-H-GluA]^-^ provided the evidence for identifying the metabolite. Thus, **D1** was tentatively characterised as glucuronidation metabolite of arctigenin. Moreover, according to literature (Gao et al., 2013), **D1** was arctigenin-4′-O-glucuronide (AG). **D2** was found at *m/z* 709.23541 with a mass of 162 Da (C_6_H_10_O_5_) more than **D1**. Moreover, **D2** was eluted at 7.06 min, and its molecular formula was inferred to be C_33_H_41_O_17_ (mass error of 0.687 ppm) in negative ion mode. Additionally, several major fragment ions at *m/z* 529 and *m/z* 371 were observed in the ESI-MS^2^ spectrum after the sequential loss of C_6_H_12_O_6_ and C_6_H_8_O_6_, respectively. And thus, **D2** was interpreted as glucuronidation and glucosylation metabolite of arctigenin.

**D4** showed the chromatographic peak at 7.59 min and possessed [M-H]^-^ ion at 451.10703 (C_21_H_23_O_9_S, mass error of 0.456 ppm), which was 80 Da more massive than arctigenin. The significant fragment ion confirmed that the product ion at *m/z* 371 was formed by the neutral loss of SO_3_. Other fragment ions at *m/z* 356, m*/z* 253, m*/z* 137, and *m/z* 67 were also observed. The results suggested that **D4** could be a sulfation metabolite of arctigenin. According to literature (Gao et al., 2013), **D4** was identified as arctigenin-4′-O-sulfate.

### Representative Metabolites of Fishhook with *m/z* 55/*m/z* 57 ([M-H]^-^/[M + H]^+^)

The characteristic fragment ion at *m/z* 55/*m/z* 57 ([M-H]^-^/[M + H]^+^) was the major fragment ion of the five-membered lactone ring. Fishing with *m/z* 55 or *m/z* 57, we found the following representative metabolites.

In negative ion mode, **E1**, eluted at 13.17 min, afforded the deprotonated molecular ion at *m/z* 401.15967 (C_22_H_25_O_7_, mass error of -0.906 ppm). It was 14 Da more massive than **A1**-**A3**, and generated fragment ions at *m/z* 383 [M-H-H_2_O]^-^, *m/z* 340 [M-H-OCH_2_-OCH_3_]^-^, and *m/z* 311 [M-H-2OCH_2_-2CH_3_]^-^, indicating that **E1** could be tentatively concluded as oxidation and methylation metabolite of arctigenin.

**E3** yield [M-H]^-^ ion at *m/z* 694.22961 (C_31_H_40_O_13_N_3_S, mass error of 0.878 ppm), which was 305 Da more massive than **C1**. In the ESI-MS^2^ spectrum, the prominent ions at *m/z* 676 [M-H-H_2_O]^-^ and *m/z* 388 [M-H-GSH]^-^ were generated, other fragment ions at *m/z* 362, m*/z* 254, m*/z* 153, and *m/z* 55 were presented. Thus, it confirmed the occurrence of internal hydrolysis and glutathione conjugation reaction.

### Representative Metabolites of Fishhook with *m/z* 39/*m/z* 41 ([M-H]^-^/[M + H]^+^)

After the five-membered lactone ring of arctigenin lost CO_2_, the fragment ion at *m/z* 39/*m/z* 41 ([M-H]^-^/[M + H]^+^) was produced. These ions were used as a bait to fish arctigenin metabolites, and many metabolites were found.

**F1** possessed theoretical [M-H]^-^ ion at *m/z* 533.16724 (C_26_H_29_O_12_, mass error of 1.483 ppm), which was 176 Da more massive than **B13**-**B15**. The ions at *m/z* 357 [M-H-GluA]^-^ and *m/z* 342 [M-H-GluA-CH_3_]^-^ provided strong evidence for our deduction in the ESI-MS^2^ spectrum. Based on the above analysis, **F1** was tentatively judged to be the demethylated and glucuronidated metabolite of arctigenin. **F12** (C_32_H_27_O_18_, mass error of -0.842 ppm) was eluted at 7.64 min and produced [M-H]^-^ ion at *m/z* 709.19794, which was 176 Da more massive than **F1**. Therefore, **F12** was preliminarily determined to be a demethylation and di-glucuronidation metabolite of arctigenin.

**F27** showed [M-H]^-^ ion at *m/z* 565.19157 (C_27_H_33_O_13_, mass error of 1.337 ppm), which was 176 Da more massive than **C1**, indicating that **F27** could be deduced as glucuronidation metabolite of **C1**. In addition, the key fragment ion at *m/z* 389 [M-H-C_6_H_8_O_6_]^-^, and other fragment ions at *m/z* 253, m*/z* 136, m*/z* 101, and *m/z* 39 were all observed in the ESI-MS^2^ spectrum. Based on mentioned above, **F27** was tentatively identified as internal hydrolysis and glucuronidation metabolite of arctigenin.

### Network pharmacology analysis

Twelve metabolites (**M0**, **B13**, **B15**, **B16**, **B19**, **B24**, **B25**, **B26**, **B28**, **C1**, **D1**, **D4**) reported in the literature were selected for network pharmacology analysis to explore the anti-AD mechanism of arctigenin.

### Related targets of arctigenin in the treatment of AD

**FIGURE 5:**
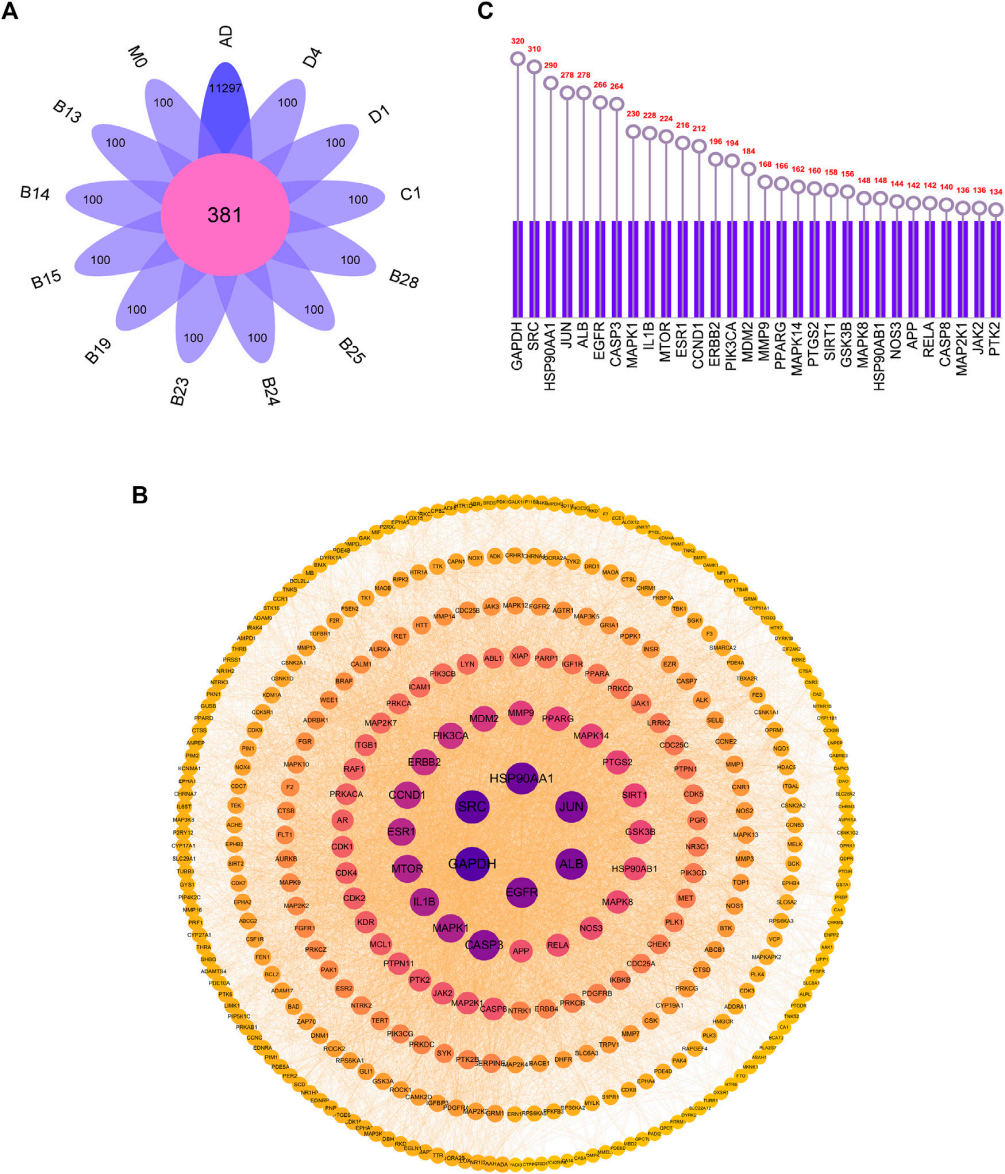
**(A)** Overlapping targets of AD-related targets and 12 metabolites targets **(B)** PPI network diagram **(C)** The top 30 core targets for arctigenin treat AD.

The targets of 12 metabolites were searched by Swiss Target Prediction database, and all targets were enriched after removing duplicate targets. AD-related targets were searched by GeneCards, and a total of 11,297 targets were retrieved, which were compared with the targets of the 12 metabolites. Subsequently, 381 common targets were determined (Figure 5A).

### Construction of the protein-protein interaction (PPI) network

All the screened targets of arctigenin against AD were imported into Cytoscape 3.9.1 software to construct PPI network diagrams. From the parameter analysis of the network nodes, there were 381 nodes and 5064 edges. Among them, the nodes with darker and larger blue were the nodes with higher degree values, indicating that they may play a more important role in the treatment of AD by arctigenin (Figure 5B). The 30 targets with the highest degree values were selected in Figure 5C, including GAPDH, SRC, HSP90AA1, JUN, ALB, *etc.*

### GO and KEGG enrichment analysis of target proteins

**FIGURE 6:**
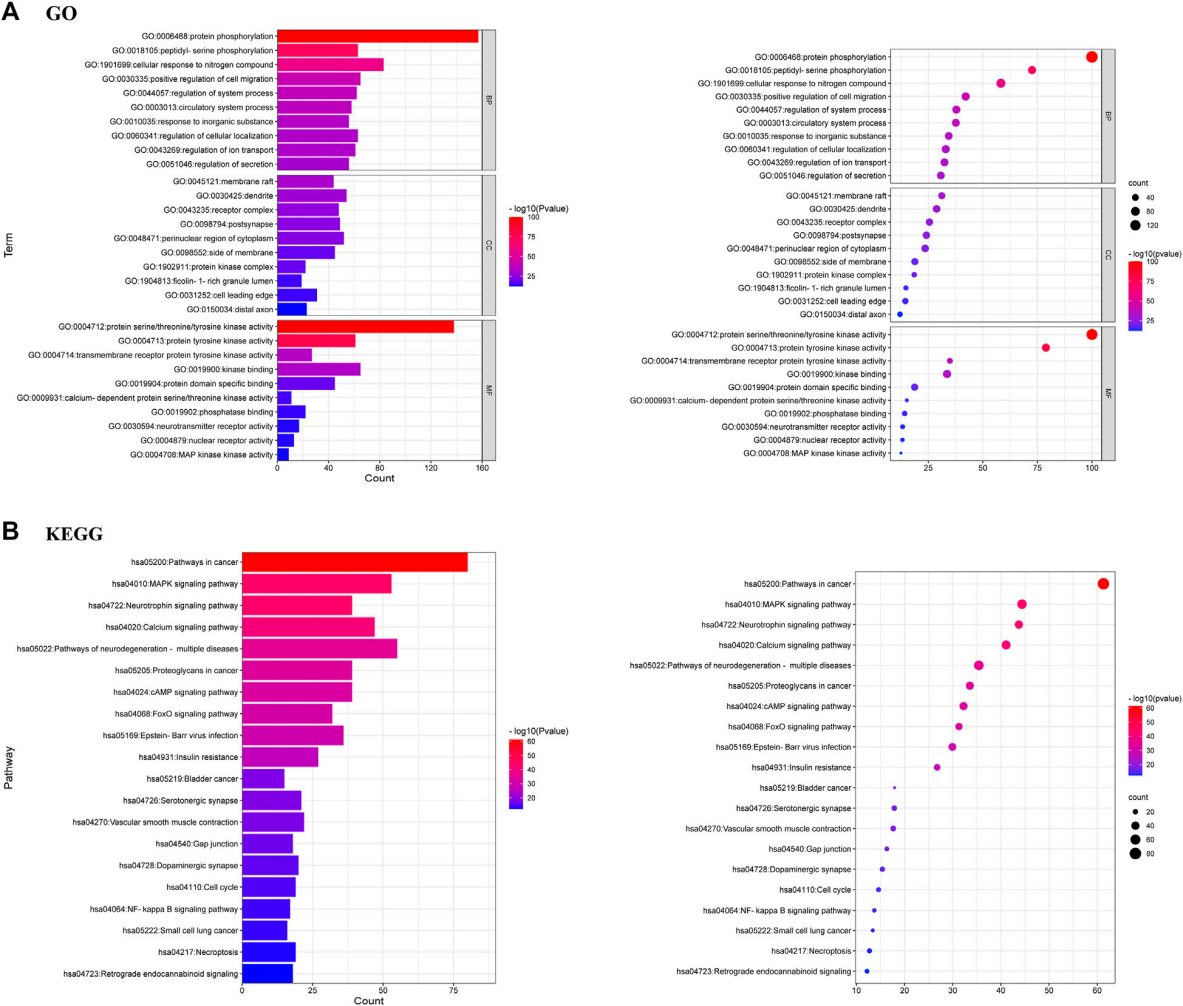
**(A)** GO analysis of 381 common targets [Biological processes (BP), Molecular functions (MF) and Cellular components (CC)] **(B)** KEGG analysis of 381 common targets.

GO enrichment analysis of 381 targets was performed by Metascape database to elucidate the biological characteristics of arctigenin for AD treatment. The top 10 enriched BP (Biological Processes), MF (Molecular Functions) and CC (Cellular Compounds) were analyzed graphically (Figure 6A; *p* < 0.01). In BP analysis, the target proteins mainly affected protein phosphorylation, peptidyl-serine phosphorylation, and cellular response to nitrogen compound. In MF analysis, target proteins were related to protein serine/threonine/tyrosine kinase activity, protein tyrosine kinase activity, and transmembrane receptor protein tyrosine kinase activity. In CC analysis, target proteins had significant effects on target protein complexes with membrane raft, dendrite, and receptors.

KEGG enrichment analysis demonstrated that the most important pathways of arctigenin metabolites for AD treatment were MAPK, Neurotrophin, Calcium, cAMP, neurodegeneration-multiple diseases, and FoxO signalling pathways. The top 20 important signaling pathways were shown in Figure 6B.

### Construction of “disease-pathways-targets-metabolites” network

**FIGURE 7:**
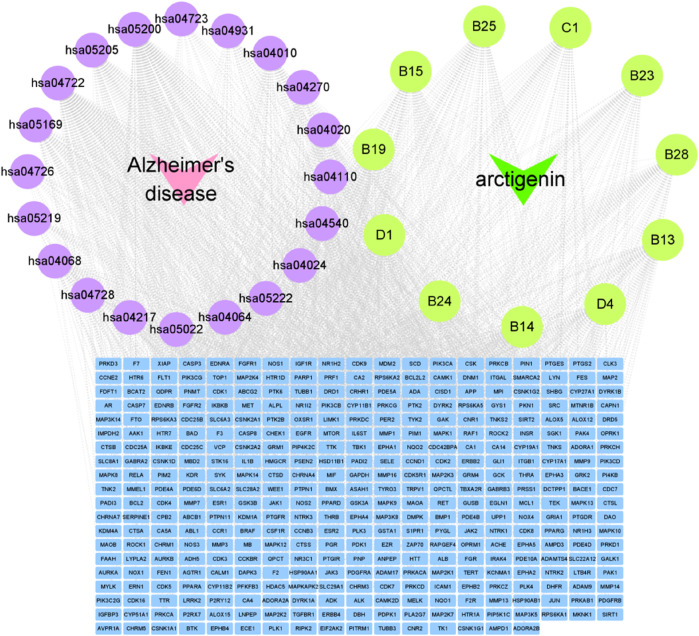
Cytoscape Network diagram (Disease-Pathway-Target-Metabolites Diagram).

The construction of the “disease-pathways-targets-metabolites” network diagram was essential for the visual analysis of arctigenin metabolites for AD treatment. Based on the above analysis results, “disease-pathways-targets-metabolites” network diagram was constructed using Cytoscape 3.9.1 software. In the diagram, the pink nodes represented AD, the purple nodes represented pathways, the green nodes represented metabolites, and the blue nodes represented targets. An edge is linked to the potential target of a metabolite (Figure 7).

## Discussion

### Selection of animal model for metabolism of arctigenin

The choice of animal model is crucial. We chose rats animal model to study the arctigenin metabolites referring to many literatures (Wu et al., 2021; Mengmeng et al., 2022; Wang Y. et al., 2022), and it was also used in the published papers of our team (Cui et al., 2022; Jing et al., 2022; Wang H. et al., 2022). There are undeniably multiple differences between human and rats. However, rodents’ unique immune system, ability to regulate liver dynamic balance, and significant genetic similarity with human make them useful tools for the study of exogenous metabolites. Studies have shown that rats animal model was capable of replicating the human-specific metabolite profiles of some drugs (Bateman et al., 2014; Scheer and Wolf, 2014). Although there are some limitations, the potential for application in drug metabolism studies is indispensable. Currently, the choice of animal model species for drug metabolism in preclinical safety assessment is not yet clear. Therefore, this also gives rise to an challenge. In addition, the overall metabolism of the drug is significant in human. These risks can be effectively assessed and mitigated if human metabolic profile of drugs can be determined in the early research stage. However, the possibility of collecting human samples through different time points is limited. These tasks cannot usually be accomplished and also involve serious ethical and technical issues.

In the present study, lots of arctigenin metabolites were found in the faeces, suggesting that the microbiota was involved in the biotransformation of metabolites. Although the differences in bacterial species and numbers in rats and human intestinal flora lead to different conversion pathways of arctigenin, our results are valuable for understanding the metabolism and excretion of arctigenin *in vivo*. It also provided useful information and references for further studies on the metabolism of arctigenin in human. Overall, the selection of rats animal model can improve the efficiency of arctigenin metabolites screening, mimic the human metabolic profile, and provide a basis for preclinical studies of arctigenin drug development.

#### Metabolic pathways of arctigenin

The retrieval and characterization of drug metabolites in biological matrices is challenging because of the interference from endogenous and exogenous components (Wang et al., 2019). Examples, include biological matrix ions, instrument noise ions, chemical reagent noise ions, pipeline pollution ions, and metabolite fragment ions, *etc.* The previously reported overall metabolite analysis methods showed that most chemical components generally maintain the basic skeleton or special structure during the metabolic process, such as the five-membered lactone ring of neolignan, which will produce a series of fragment ions with diagnostic function in LC-MS analysis (Borbély et al., 2016). Therefore, we described a Serial Five-Membered Lactone Ring Ions Fishing-based data-mining method for the identification of arctigenin metabolites.

Only 11 metabolites including **B13**, **B14**, **B15**, **B19**, **B23**, **B24**, **B25**, **B28**, **C1**, **D1**, and **D4** were detected in the previous studies. Based on serial five-membered lactone ring ions fishing strategy, 94 new arctigenin metabolites were discovered in this study. The results characterized 76 metabolites in rats and 49 metabolites in liver microsomes. Most of arctigenin metabolites were detected in rat urine and faeces. Previously, it was reported that increased gut and blood-brain barrier permeability due to microbiota dysbiosis may mediate the pathogenesis of AD (Gegunde et al., 2021; Shoubridge et al., 2021; Sun et al., 2021). Based on the metabolic results, it was speculated that arctigenin could produce many metabolites under the transformation of intestinal microbiota, which may play a potential role in the treatment of AD. At the same time, many metabolites could also be detected in liver microsomes experiment *in vitro*. Therefore, the combined study of arctigenin metabolites *in vivo* and *in vitro* could provide strong support for treating AD. The metabolic profile of arctigenin was shown in Figure 4. These metabolic reactions mainly include the major phase I reactions of oxidation, reduction, and internal hydrolysis, and the major phase II reactions of glucuronidation, sulfation, methylation, acetylcysteine conjugation and glutathione binding, and their composite reactions. Among them, many fat-soluble metabolites were found, which were beneficial to exerting efficacy through the blood-brain barrier (Fukuyama, 2022; Trushina et al., 2022).

Furthermore, arctigenic acid was a very important arctigenin metabolite. Many arctigenic acid metabolites have been found by *m/z* 101 ([M-H]^-^)/*m/z* 103 ([M + H]^+^) ion fishing, indicating that arctigenic acid has further metabolic reaction as arctigenin secondary metabolite. As a result, after oral administration of arctigenin to rats, it was not detected in plasma, but its newly identified metabolite arctigenic acid could be detected in plasma. Therefore, arctigenic acid can be used as a monitoring indicator after oral administration of arctigenin or products and supplements containing burdock for AD.

In light of the results, phase I and phase II metabolites were obtained by fishing a series of five-membered lactone ring fragments. The fragment ions of metabolites confirmed that the debenzylation cleavage pathway was a common pathway. A series of five-membered lactone ring fragment ions were considered the characteristic ions of arctigenin metabolites. Referring to compounds with similar structural characteristics will facilitate the comprehensive identification of arctigenin metabolites.

### Comparison of arctigenin metabolism *in vivo* and *in vitro*

Biotransformation plays an important role in drug metabolism fields. Current studies on drug metabolism have focused on biological samples from rats. In addition, *in vitro* incubation with liver microsomes is gradually coming to the forefront, and these studies demonstrate that it expands the study of drug metabolites. (Kasper et al., 1994; Jin et al., 2007; Gao et al., 2013). Herein, 76 metabolites in rats (26 in plasma, 53 in urine, 42 in faeces, and 11 in liver), 49 metabolites in liver microsomes were investigated. *In vivo* metabolite studies, most of arctigenin metabolites were excreted through urine and faeces. This implied that the metabolic activity of rat urine and faeces samples was higher than plasma and liver, and also indicated that both were powerful terms to study arctigenin metabolites. It was not difficult to find that although the liver is the most important metabolic organ in the body, few metabolites were obtained from the liver and most of them were glucuronidation products and sulfation products of arctigenin. Notably, arctigenin acid, an important metabolite, was only obtained in plasma samples, which also provided a basis for the conversion of arctigenin into blood components. *In vitro*, liver microsomes also showed strong metabolism, and although few arctigenin metabolites were found in liver microsomes than in rats, most phase II metabolites were found in liver microsomes, and 31 arctigenin metabolites were detected only in liver microsomes. In general, the *in vivo* biotransformation of arctigenin was much more complicated than *in vitro*, but 24 metabolic changes such as glutathionylation, debenzylation, loss of O, the continuous reactions of demethylation and so on exist *in vitro*. This method enriched the deficiency of *in vivo* biotransformation and provided a valuable reference for the study of arctigenin metabolites.

### Network pharmacology analysis of twelve metabolites

Alzheimer’s disease (AD) is a progressive neurodegenerative disease with pathogenesis concealment. It is characterized clinically by a comprehensive spectrum of dementia manifesting as memory impairment, aphasia, dysfluency, dyscognition, impairment of visuospatial skills, executive dysfunction, and personality and behavioral changes (Cleland et al., 2021). Studies have shown that increased β Amyloid (Aβ) may contribute to the pathogenesis of AD, as the continued accumulation of the protein in the hippocampus and cortex leads to cognitive deficits and neuronal dysfunction (Qi et al., 2017). Therefore, drugs with dual functions of Aβ inhibition and clearance may show more effective anti-AD properties. Considering that natural products are the main source of bioactive agents due to their structural diversity, arctigenin was found in the natural product library (Zhu et al., 2013). However, how this influence is precisely working is yet to be discovered.

Based on previous studies of arctigenin metabolites, network pharmacology was used to predict the targets and potential pathways of 12 metabolites for the treatment of AD. According to the target protein interaction network, a total of 30 core targets were screened out, including GAPDH, SRC, HSP90AA1, JUN, ALB, *etc.* Among them, GAPDH was used as a promising therapeutic target. Researchers claim that large amounts of cytoplasmic proteins are released due to the death of neuronal cells in the late stages of AD (Kunkle et al., 2019). GAPDH, one of the cytoplasmic proteins, forms stable conjugates with extracellular Aβ, the levels of which are positively correlated with disease progression in AD patients. These binders can also lead to cognitive impairment (Wang et al., 2005). In addition, it was also found that lentivirus-driven overexpression of GAPDH in AD models increased levels of hippocampal apoptosis, neuronal degeneration, and cognitive dysfunction (Wilhelmus et al., 2014). In contrast, knockout of the GAPDH gene in mice reversed neuropathic lesions, suggesting that GAPDH plays a pivotal role in Aβ-stimulated AD (Lazarev et al., 2021).

Furthermore, GO and KEGG enrichment analyses suggest that the MAPK pathway may be the predominant pathway that plays a role in the treatment of AD by 12 metabolites. Several studies have revealed that Aβ is a pathological hallmark of AD (Ji et al., 2016). Aβ activation of p38 MAPK leads to increased intracellular calcium, mitochondrial stress, and ROS production, all of which responses exacerbate the pathogenesis of AD (Calkins and Reddy, 2011; Lee and Kim, 2017). In addition, JUN is a key molecular target selected from the PPI networks for treating AD and is also thought to be an apoptotic transcription factor leading to neuronal cell death in AD. *c*-JUN, a downstream target, can be upregulated by p38 MAPK, which in turn leads to neuronal cell death (Chang et al., 2010). *In vitro* experimental studies have shown that exposure to Aβ in primary cultured neurons and immortalized neuronal cells can activate p38 MAPK and promote neuronal apoptosis in AD patients (Chen et al., 2016; Suwanna et al., 2014).

Although we have predicted the target and pathway of twelve metabolites in the treatment of AD through network pharmacology, bioinformatics methods still have some limitations. We found that using network pharmacology alone to explore the efficacy of arctigenin metabolites for treating AD was too one-sided. Further pharmacological experiments and systematic molecular biology experiments are needed to verify the exact mechanism.

## Conclusion

In this study, a strategy based on the combination of ions fishing and multiple MS acquisition data processing methods was established. The practicality and effectiveness of the ions fishing-based analysis strategy was verified by analyzing the arctigenin metabolites for the first time. The results characterized 76 metabolites in rats and 49 metabolites in liver microsomes, indicating that arctigenin undergoes extensive phase I and phase II metabolism after entering the biological system, including oxidation, reduction, hydrolysis, and complexation reactions. Additionally, 381 common targets of disease-metabolites were revealed *via* network pharmacology. Among them, the five key targets with the highest degree values were GAPDH, SRC, HSP90AA1, JUN, and ALB, which play significant roles in the treatment of AD mainly through MAPK, Neurotrophin, Calcium, cAMP, neurodegeneration-multiple diseases, FoxO signaling pathways. In brief, our research results not only established an analytical strategy for the rapid discovery and identification of metabolites *in vivo*, but also provided references for the therapeutic and application of arctigenin in Alzheimer’s disease and other neurodegenerative diseases.

## Data Availability

The original contributions presented in the study are included in the article/Supplementary Material further inquiries can be directed to the corresponding authors.
