# Supplementary material for: Serial five-membered lactone ring ions in the treatment of Alzheimer’s diseases-comprehensive profiling of arctigenin metabolites and network analysis
Source: Front Pharmacol. 2022 Dec 20;13:1065654. doi: 10.3389/fphar.2022.1065654 (PMC9807626; doi:10.3389/fphar.2022.1065654)
Supplement: Supplementary file 1 [file Table1.docx]

**TABLE S1 |** Summary of Arctigenin Metabolites *in vivo* and *in vitro*

| **ID** | **Identification/ Reactions** | **t_R_/**  **min** | **Ion**  **Mode** | **Formula**   1. **H]^-^/**   **[M+H]^+^** | **Theoretical**  **Mass (*m/z*)** | **Experimental**  **Mass (*m/z*)** | **Error**  **(ppm)** | **RDB** | **MS/MS Fragment Ions** | **PS** | **PM** | **PA** | **U** | **F** | **L** | **LM** |
| --- | --- | --- | --- | --- | --- | --- | --- | --- | --- | --- | --- | --- | --- | --- | --- | --- |
| **M0*** | arctigenin | 9.84 | N | C_21_ H_23_ O_6_ | 371.14891 | 371.15067 | 0.774 | 10.5 | **83(100.00)**,371(64.40),136(47.84),121(12.23),356(7.09),235(3.64),137(3.02),249(2.22),55(2.17),357(1.31),97(1.05),216(0.63) | - | - | - | + | - | - | - |
|  |  | 9.85 | P | C_21_ H_25_ O_6_ | 373.16457 | 373.16364 | -0.479 | 9.5 | 137(100.00),373(8.73),177(8.59),151(8.58),305(6.25),323(3.08),337(2.69),356(1.65),189(0.92),217(0.76),263(0.63),245(0.55),175(0.46),203(0.38) | - | - | - | + | - | - | + |
| **A1** | oxidation | 6.70 | N | C_21_H_23_O_7_ | 387.14383 | 387.14868 | 3.754 | 10.5 | 387(100.00),113(36.96),**83(22.80)**,211(19.29),124(7.23),235(4.92) | - | - | - | + | - | - | + |
| **A2** | oxidation | 8.38 | N | C_21_H_23_O_7_ | 387.14383 | 387.14352 | -3.632 | 10.5 | 387(100.00),**83(69.76)**,151(31.81),137(30.64),372(12.99) | - | - | - | - | + | - | + |
|  |  | 8.40 | P | C_21_H_25_O_7_ | 389.15948 | 389.15854 | -2.414 | 9.5 | 153(100.00),371(36.73),151(25.70),389(17.84),121(7.47),321(5.33) | - | - | - | - | - | - | + |
| **A3** | oxidation | 8.45 | N | C_21_H_23_O_7_ | 387.14383 | 387.14532 | 1.017 | 10.5 | 387(100.00),137(80.61),**83(58.18)**,151(34.08),121(3.43),357(1.78) | - | - | - | - | - | - | + |
| **A4** | demethylation and oxidation | 7.08 | N | C_20_H_21_O_7_ | 373.12818 | 373.12985 | 1.538 | 10.5 | 329(100.00),373(64.76),284(44.24),**83(18.79)**,121(11.36),136(10.64) | - | + | + | + | + | - | + |
| **A5** | dioxidation | 6.22 | N | C_21_H_23_O_8_ | 403.13874 | 403.13943 | -1.019 | 10.5 | 233(100.00),403(59.50),137(40.94),139(40.75),151(25.78),**83(15.88)** | - | - | - | - | - | - | + |
|  |  | 6.21 | P | C_21_H_25_O_8_ | 405.15439 | 405.15439 | -0.010 | 9.5 | 153(100.00),167(72.96),387(27.18),337(12.92),405(9.42) | - | - | - | - | - | - | + |
| **A6** | tri-oxidation | 6.96 | N | C_21_H_23_O_9_ | 419.13366 | 419.13516 | 0.965 | 10.5 | 419(100.00),243(55.36),**83(33.52)**,123(6.64),252(2.48),352(2.39) | + | + | + | - | - | - | - |
| **A7** | hydroxylation and dehydration | 9.65 | N | C_21_H_21_O_6_ | 369.13326 | 369.13470 | 0.917 | 11.5 | 369(100.00),174(34.92),**83(28.75)**,218(7.62),354(6.33),235(1.25) | - | - | - | - | - | - | + |
| **B1** | dihydrogenation | 9.13 | N | C_21_H_27_O_6_ | 375.18022 | 375.18463 | 3.498 | 8.5 | 375(54.60),293(46.69),**57(13.22)**,329(0.95),277(0.56),344(0.50) | - | - | - | - | + | - | - |
|  |  | 9.16 | P | C_21_H_29_O_6_ | 377.19587 | 377.19461 | -3.327 | 7.5 | 377(85.30),275(27.32),317(20.85),361(11.57),281(8.80),243(7.03) | - | - | - | + | - | - | - |
| **B2** | dihydrogenation | 9.31 | N | C_21_H_27_O_6_ | 375.18022 | 375.18478 | 3.468 | 8.5 | **57(100.00)**,375(81.44),293(51.26),113(3.10),277(2.97),297(1.82) | - | - | - | - | + | - | - |
| **B3** | dihydrogenation | 9.50 | N | C_21_H_27_O_6_ | 375.18022 | 375.18481 | 3.498 | 8.5 | 375(100.00),**57(32.93)**,293(20.56),191(14.85),357(4.71),297(0.61) | - | - | - | - | + | - | - |
| **B4** | dihydrogenation | 9.68 | N | C_21_H_27_O_6_ | 375.18022 | 375.18481 | 3.498 | 8.5 | 375(100.00),295(21.69),96(14.44),137(6.41),**57(6.31)** | - | - | - | - | + | - | - |
| **B5** | dihydrogenation | 9.86 | N | C_21_H_27_O_6_ | 375.18022 | 375.18469 | 3.378 | 8.5 | 79(100.00),375(93.76),191(35.04),277(7.13),357(3.70),297(1.49),**83(1.04)** | - | - | - | - | + | - | - |
| **B6** | tri-hydrogenation | 8.77 | N | C_21_H_29_O_6_ | 377.19587 | 377.19833 | 1.386 | 7.5 | 377(56.32),297(19.09),**57(3.94)**,344(1.33),329(0.93) | - | - | - | - | + | - | - |
|  |  | 8.73 | P | C_21_H_31_O_6_ | 379.21152 | 379.21185 | 0.883 | 6.5 | 379(100.00),123(33.44),95(30.20),**85(30.20)**,343(15.87),359(15.02) | - | - | - | - | + | - | - |
| **B7** | dihydrogenation and dehydroxylation | 8.42 | N | C_21_H_27_O_5_ | 359.18530 | 359.18665 | 0.704 | 8.5 | 359(100.00),315(20.45),121(6.29),344(5.52),221(5.33),**57(3.42)** | - | - | - | + | - | - | - |
|  |  | 8.41 | P | C_21_H_29_O_5_ | 361.20095 | 361.20013 | -2.272 | 7.5 | 361(100.00),315(29.80),297(23.27),279(17.33),261(6.70),**59(5.40)** | + | + | + | + | + | + | + |
| **B8** | hydrogenation and decarbonylation | 10.25 | N | C_20_H_25_O_5_ | 345.16965 | 345.17047 | -0.803 | 8.5 | 345(100.00),257(37.12),300(30.78),137(10.03),**57(9.08)**,283(7.76) | - | - | - | + | - | - | - |
|  |  | 10.22 | P | C_20_H_27_O_5_ | 347.18530 | 347.18292 | -6.856 | 7.5 | 347(100.00),283(84.68),**59(65.24)**,329(63.54),123(60.24),135(40.30) | - | - | - | + | - | - | - |
| **B9** | hydrogenation,  decarbonylation, and demethylation | 8.67 | N | C_19_H_23_O_5_ | 331.15400 | 331.15546 | 0.363 | 8.5 | 331(100.00),113(25.12),155(22.47),287(21.83),**57(18.19)**,121(3.18) | - | - | - | + | + | - | - |
| **B10** | dihydrogenation and  demethylation | 11.56 | N | C_20_H_25_O_6_ | 361.16457 | 361.16409 | -4.352 | 8.5 | 236(100.00),221(99.63),302(6.82),**57(4.45)**,**83(3.99)**,273(3.97) | + | + | + | + | - | - | - |
| **B11** | decarbonylation | 13.67 | N | C_20_H_23_O_5_ | 343.15400 | 343.15491 | -0.187 | 9.5 | 343(100.00),315(79.26),**57(18.89)**,316(12.32),286(10.90),299(7.10),136(2.47) | - | - | - | + | - | - | - |
| **B12** | demethylation,  dihydroxylation, and  hydrogenation | 6.50 | N | C_20_H_23_O_8_ | 391.13874 | 391.14050 | 0.659 | 9.5 | 391(100.00),329(75.57),177(20.78),315(20.37),343(7.38),**57(5.21)** | - | - | - | - | - | - | + |
| **B13** | Matairesinol | 8.50 | N | C_20_H_21_O_6_ | 357.13326 | 357.13321 | -3.225 | 10.5 | 357(100.00),**57(57.16)**,121(26.06),136(21.43),221(4.21),**83(0.71)** | - | - | - | + | + | - | + |
|  |  | 8.50 | P | C_20_H_23_O_6_ | 359.14719 | 359.14761 | -3.633 | 9.5 | 137(100.00),341(11.14),**59(7.81)**,359(6.11),223(1.84),123(1.77) | - | - | - | + | + | - | + |
| **B14** | 3”-O-demethyl-arctigenin | 8.68 | N | C_20_H_21_O_6_ | 357.13326 | 357.13550 | 3.188 | 10.5 | 357(100.00),**57(76.36)**,121(33.02),137(4.72) | - | - | - | + | + | - | + |
|  |  | 8.68 | P | C_20_H_23_O_6_ | 359.14719 | 359.14880 | -3.466 | 9.5 | 123(100.00),137(55.18),**85(46.96)**,359(31.55),341(16.65),**59(15.47)** | - | - | - | + | + | - | - |
| **B15** | 3’-O-demethyl-arctigenin | 8.85 | N | C_20_H_21_O_6_ | 357.13326 | 357.13535 | 2.767 | 10.5 | 357(100.00),**57(57.16)**,122(34.84),137(8.56) | - | - | - | + | + | - | + |
|  |  | 8.86 | P | C_20_H_23_O_6_ | 359.14719 | 359.14891 | -4.803 | 9.5 | 137(100.00),341(11.24),359(8.33),131(6.76),323(3.56),**59(3.00)** | - | - | - | + | + | - | + |
| **B16** | demethylation and dehydroxylation | 9.88 | N | C_20_H_21_O_5_ | 341.13835 | 341.13947 | 0.067 | 10.5 | 341(100.00),326(34.11),121(25.74),137(14.47),297(1.92),**57(1.09)** | - | - | - | + | + | - | - |
|  |  | 9.86 | P | C_20_H_23_O_5_ | 343.15400 | 343.15298 | -2.973 | 9.5 | 151(100.00),343(74.77),325(34.31),275(30.09),121(24.50),**59(11.09)** | - | - | - | + | + | - | - |
| **B17** | demethylation and  dihydroxylation | 5.64 | N | C_20_H_21_O_8_ | 389.12309 | 389.12473 | 0.539 | 10.5 | 389(100.00),371(14.20),356(11.45),153(9.31),341(5.70),**57(4.02)** | - | - | - | - | - | - | + |
| **B18** | demethylation and tri-hydroxylation | 5.82 | N | C_20_H_21_O_9_ | 405.11801 | 405.11963 | 1.295 | 10.5 | 113(100.00),405(73.79),175(55.42),229(53.97),**57(44.30)**,123(40.41) | - | + | - | - | - | - | - |
| **B19** | enterolactone | 8.88 | N | C_18_H_17_O_4_ | 297.11214 | 297.11346 | 0.766 | 10.5 | 297(100.00),253(33.93),107(33.78),134(18.81),121(13.64),**57(7.80)** | + | + | + | + | + | + | - |
|  |  | 8.86 | P | C_18_H_19_O_4_ | 299.12779 | 299.12802 | 0.784 | 9.5 | 133(100.00),263(19.78),299(18.89),245(7.98),121(5.82) | - | + | + | + | + | - | - |
| **B20** | demethylation and  decarbonylation | 6.78 | N | C_19_H_21_O_5_ | 329.13835 | 329.13940 | -0.047 | 9.5 | 329(100.00),178(35.71),**57(14.32)**,150(13.39),316(7.00),121(5.03) | - | - | - | - | - | - | + |
| **B21** | demethylation and dehydrogenation | 8.44 | N | C_20_H_19_O_6_ | 355.11761 | 355.11893 | 0.218 | 11.5 | 355(100.00),340(38.38),256(16.27),**57(14.81)**,325(12.75) | - | - | - | - | - | - | + |
|  |  | 8.43 | P | C_20_H_21_O_6_ | 357.13326 | 357.13242 | -0.845 | 10.5 | 357(100.00),137(96.02),233(54.97),129(51.29),201(40.71) | - | - | - | - | - | - | + |
| **B22** | demethylation and  methylene ketylation | 8.56 | N | C_20_H_19_O_7_ | 371.11253 | 371.11349 | -0.136 | 11.5 | 371(100.00),**57(44.64)**,327(19.74),312(17.69),151(15.58),357(9.20) | - | - | - | - | - | - | + |
| **B23** | didemethylation | 7.72 | N | C_19_H_19_O_6_ | 343.11761 | 343.11960 | 2.589 | 10.5 | 343(100.00),122(55.13),**57(47.03)**,137(15.56),229(14.60),221(9.79) | - | - | - | + | + | - | + |
|  |  | 7.72 | P | C_19_H_21_O_6_ | 345.13326 | 345.13275 | -1.492 | 9.5 | 123(100.00),137(16.91),223(16.85),345(16.24),327(15.83),309(6.86) | - | - | - | + | + | - | + |
| **B24** | didemethylation | 7.58 | N | C_19_H_19_O_6_ | 343.11761 | 343.11957 | 2.502 | 10.5 | 79(58.61),343(44.60),**57(25.21)**,219(21.57),149(15.15),121(8.10) | - | - | - | + | + | - | + |
|  |  | 7.61 | P | C_19_H_21_O_6_ | 345.13326 | 345.13199 | -3.694 | 9.5 | 123(100.00),137(40.48),327(17.29),223(13.88),345(12.65) | - | - | - | - | - | - | + |
| **B25** | didemethylation | 7.43 | N | C_19_H_19_O_6_ | 343.11761 | 343.11880 | 0.258 | 10.5 | 343(100.00),**57(34.36)**,121(13.39),137(8.86),221(5.27) | - | - | - | + | + | - | + |
|  |  | 7.43 | P | C_19_H_21_O_6_ | 345.13326 | 345.13245 | -2.361 | 9.5 | 137(100.00),327(12.33),123(10.60),**59(10.55)**,345(6.71) | - | - | - | + | + | - | + |
| **B26** | didemethylation and dehydroxylation | 8.74 | N | C_19_H_19_O_5_ | 327.12270 | 327.12402 | 0.682 | 10.5 | 327(100.00),123(24.98),242(20.55),147(13.93),**57(4.64)**,312(4.37) | - | + | + | + | - | + | - |
|  |  | 8.71 | P | C_19_H_21_O_5_ | 329.13835 | 329.13773 | -1.884 | 9.5 | 137(100.00),329(15.59),233(4.53),293(4.45),132(4.35) | - | - | - | + | + | - | - |
| **B27** | 3’,3”,4”-O-tri-demethyl-arctigenin | 6.47 | N | C_18_H_17_O_6_ | 329.10196 | 329.10333 | 0.178 | 10.5 | 329(100.00),311(23.81),285(14.37),121(13.35),135(7.17),**57(3.16)** | - | + | + | + | + | - | + |
|  |  | 6.49 | P | C_18_H_19_O_6_ | 331.11761 | 331.11719 | -1.283 | 9.5 | 123(100.00),313(13.99),331(8.40),295(6.59),**59(5.03)** | - | - | - | + | + | - | - |
| **B28** | 4’,4”-dihydroxy-enterolactone | 7.73 | N | C_18_H_17_O_6_ | 329.10196 | 329.10330 | 0.725 | 10.5 | 329(100.00),311(18.92),137(9.94)285(1.91),121(1.91),**57(1.01)** | - | - | - | + | + | - | - |
| **B29** | tri-demethylation and dehydroxylation | 7.56 | N | C_18_H_17_O_5_ | 313.10705 | 313.10846 | 1.000 | 10.5 | 313(100.00),269(28.94),123(25.65),**57(2.08)** | - | - | - | + | + | - | - |
|  |  | 7.57 | P | C_18_H_19_O_5_ | 315.12270 | 315.12210 | -1.905 | 9.5 | **59(100.00)**,149(65.54),123(58.10),297(43.61),279(30.41),315(28.57) | - | - | - | + | + | - | - |
| **B30** | dehydroxylation and demethoxy | 7.83 | P | C_20_H_23_O_4_ | 327.15909 | 327.15906 | -0.079 | 9.5 | 327(100.00),263(61.34),239(9.21)137(6.09),107(5.91),**59(4.31)** | - | - | - | + | - | - | - |
| **B31** | dehydroxylation and tri-demethoxy | 7.32 | P | C_18_H_19_O_2_ | 267.13796 | 267.13873 | 2.896 | 9.5 | 107(100.00),**59(32.69)**,267(30.78),147(17.89),121(9.69),135(4.38) | - | - | - | - | + | - | - |
| **B32** | demethylation(B-ring) and debenzylation(A-ring) | 8.13 | N | C_12_H_13_O_4_ | 221.08084 | 221.08194 | 0.008 | 6.5 | 177(100.00),221(38.76),136(6.62),124(6.49),**57(3.33)** | - | - | - | + | + | - | - |
|  |  | 8.14 | P | C_12_H_15_O_4_ | 223.09649 | 223.09601 | -0.475 | 5.5 | 145(100.00),205(87.16),223(69.14),177(67.33),137(23.24),**59(13.37)** | - | - | - | - | - | - | + |
| **B33** | debenzylation(B-ring) | 9.94 | N | C_12_H_13_O_4_ | 221.08084 | 221.08168 | -1.141 | 6.5 | **57(100.00)**,121(51.37),221(47.29),177(14.62),204(5.79) | + | + | + | + | + | + | + |
|  |  | 9.98 | P | C_12_H_15_O_4_ | 223.09649 | 223.09618 | -1.369 | 5.5 | 145(100.00),177(88.66),223(70.43),**59(29.29)**,137(19.18) | - | - | - | - | - | - | + |
| **B34** | debenzylation(A-ring) | 10.24 | N | C_13_H_15_O_4_ | 235.09649 | 235.09700 | -2.477 | 6.5 | 103(100.00),146(51.12),123(29.06),235(10.09),220(3.60),**57(2.59)** | + | + | - | + | - | - | + |
|  |  | 10.27 | P | C_13_H_17_O_4_ | 237.11213 | 237.11194 | -0.825 | 5.5 | 191(100.00),219(75.68),237(63.75),**59(31.15)**,137(10.16) | - | - | - | + | + | - | + |
| **B35** | hydrogenation and dihydroxylation | 6.64 | N | C_21_H_25_O_8_ | 405.15439 | 405.15610 | 1.503 | 9.5 | 405(100.00),137(47.41),346(25.44),253(19.46),**57(7.45)**,123(4.23) | - | - | - | - | - | - | + |
| **C1** | arctigenic acid | 7.43 | N | C_21_H_25_O_7_ | 389.15948 | 389.16119 | 1.577 | 9.5 | 389(100.00),330(54.74),121(26.01),**101(17.60)**,345(6.37),253(0.78) | - | + | + | + | + | - | + |
|  |  | 7.49 | P | C_21_H_27_O_7_ | 391.17513 | 391.17572 | 1.509 | 8.5 | 159(100.00),245(43.67),170(25.62),374(21.09),**103(10.01)** | + | - | - | - | - | - | - |
| **C2** | internal bis-hydrolysis | 6.72 | N | C_21_H_27_O_8_ | 407.17004 | 407.17111 | -0.076 | 8.5 | **101(100.00)**,407(81.99),325(36.16),241(24.07),59(23.56),124(20.83) | - | - | - | - | + | - | - |
| **C3** | internal hydrolysis and demethylation | 6.51 | N | C_20_H_23_O_7_ | 375.14383 | 375.14508 | 0.410 | 9.5 | 375(100.00),316(28.27),219(17.84),331(11.40),121(10.22),**101(4.04)** | - | + | + | - | + | - | + |
| **C4** | internal hydrolysis and demethylation | 6.85 | N | C_20_H_23_O_7_ | 375.14383 | 375.14511 | 0.184 | 9.5 | 375(100.00),122(23.36),316(21.34),121(17.29),**101(11.60)**,331(10.14) | - | - | - | - | - | - | + |
| **C5** | internal hydrolysis and  bis-demethylation | 5.77 | N | C_19_H_21_O_7_ | 361.12818 | 361.12976 | 1.340 | 9.5 | 361(100.00),**101(33.74)**,239(20.86),317(13.81),343(7.04) | - | - | - | - | - | - | + |
| **C6** | internal hydrolysis,  bis-demethylation, and demethoxy | 8.34 | N | C_18_H_19_O_6_ | 331.11761 | 331.11798 | -2.209 | 9.5 | 287(100.00),275(44.11),331(37.59),**101(23.00)**,243(19.42) | - | - | - | + | - | - | - |
|  |  | 8.39 | P | C_18_H_21_O_6_ | 333.13326 | 333.13266 | -1.816 | 8.5 | **103(100.00)**,287(72.30),305(72.10),333(62.23),315(36.34) | - | - | - | - | - | - | + |
| **C7** | internal hydrolysis,  bis-demethylation, demethoxy,  and dehydroxylation | 7.07 | N | C_18_H_19_O_5_ | 315.12270 | 315.12433 | 1.691 | 9.5 | 315(100.00),271(96.09),137(19.91),297(15.70),253(15.02),**101(10.09)** | + | + | + | + | - | + | - |
| **C8** | internal hydrolysis and  tri-demethylation | 7.74 | N | C_18_H_19_O_7_ | 347.11253 | 347.11356 | -0.191 | 9.5 | 347(100.00),345(97.60),122(46.38),136(32.72),**101(20.27)** | - | - | - | - | - | - | + |
|  |  | 7.75 | P | C_18_H_21_O_7_ | 349.12818 | 349.12732 | -2.462 | 8.5 | 233(100.00),136(63.51),313(52.87),349(50.41),123(49.22),**103(33.12)** | - | - | - | - | - | - | + |
| **C9** | internal hydrolysis and dihydroxylation | 7.99 | N | C_21_H_25_O_9_ | 421.14931 | 421.15057 | 0.390 | 9.5 | 245(100.00),113(92.32),**101(44.04)**,421(35.72),343(30.51)121(4.90),373(4.81),221(3.89),137(3.84) | - | - | - | + | - | - | - |
| **C10** | internal hydrolysis and  demethoxy | 7.74 | N | C_20_H_23_O_6_ | 359.14891 | 359.15060 | 1.638 | 9.5 | 359(100.00),121(83.65),315(10.37),136(8.35),122(5.16),**101(4.78)** | - | - | - | - | + | - | - |
| **C11** | internal hydrolysis, demethoxy,  and dehydroxylation | 13.67 | N | C_20_H_23_O_5_ | 343.15400 | 343.15491 | -0.545 | 9.5 | 343(100.00),315(79.26),271(15.17),286(10.90)306(8.02),**101(5.07)** | - | - | - | + | - | - | - |
| **C12** | internal hydrolysis and  debenzylation(A-ring) | 8.46 | N | C_13_H_17_O_5_ | 253.10705 | 253.10840 | 1.000 | 5.5 | 217(100.00),253(89.55),191(12.91),235(9.66),**101(7.88)** | - | + | + | + | - | - | - |
| **C13** | internal hydrolysis and didemethoxy | 5.33 | N | C_19_H_21_O_5_ | 329.13835 | 329.13934 | -0.325 | 9.5 | 329(100.00),314(71.30),299(34.80),284(29.58),124(6.09),**101(3.67)** | - | - | - | + | - | - | + |
| **C14** | internal hydrolysis and  debenzylation(B-ring) | 7.59 | N | C_12_H_15_O_5_ | 239.09140 | 239.09236 | -0.572 | 5.5 | 239(100.00),183(35.49),195(11.21),**101(10.29)**,121(4.57) | - | + | + | + | - | - | - |
| **C15** | internal hydrolysis  and decarbonylation | 11.56 | N | C_20_H_25_O_6_ | 361.16457 | 361.16409 | -1.572 | 8.5 | **101(100.00)**,221(99.63),302(6.82),327(4.45),330(2.94) | + | + | + | + | - | - | - |
| **C16** | internal hydrolysis and decarboxylation | 10.25 | N | C_20_H_25_O_5_ | 345.16965 | 345.17047 | -0.803 | 8.5 | 345(100.00),257(37.12),300(30.78),256(15.79),137(10.03),**101(9.24)** | - | - | - | + | - | - | - |
| **C17** | internal hydrolysis,  dihydrogenation | 10.38 | N | C_21_H_29_O_7_ | 393.19078 | 393.19196 | 0.212 | 7.5 | 393(66.68),331(5.54),347(2.36),329(1.68),295(1.80),**101(0.57)** | - | - | - | - | + | - | - |
| **C18** | internal hydrolysis,  decarbonylation, and hydrogenation | 8.99 | N | C_20_H_27_O_6_ | 363.18022 | 363.18124 | -0.198 | 7.5 | 363(100.00),301(53.55),345(13.69),320(6.43),315(3.26),**101(2.19)** | - | - | - | - | + | - | - |
| **D1** | arctigenin-4’-O-glucuronide | 7.32 | N | C_27_H_31_O_12_ | 547.18100 | 547.18286 | 1.390 | 12.5 | 547(100.00),113(77.23),**67(61.95)**,136(17.00),371(13.74) | + | + | + | + | - | + | + |
| **D2** | glucuronidation and  glucosylation | 7.06 | N | C_33_H_41_O_17_ | 709.23383 | 709.23541 | 0.687 | 13.5 | 59(100.00),709(53.68),**83(17.88)**,529(15.24),371(9.09),**67(3.21)** | - | - | - | - | + | - | - |
| **D3** | diglucuronidation | 8.29 | N | C_33_H_39_O_18_ | 723.21309 | 723.21710 | -0.826 | 14.5 | 417(100.00),387(43.84),359(40.28),**67(14.47)**,723(7.55),372(5.46) | - | - | - | - | - | - | + |
|  |  | 8.29 | P | C_33_H_41_O_18_ | 725.22874 | 725.22760 | -1.573 | 13.5 | 419(100.00),389(57.56),725(5.17),371(5.00),**69(3.41)**,343(2.62) | - | - | - | - | - | - | + |
| **D4** | arctigenin-4’-O-sulfation | 7.59 | N | C_21_H_23_O_9_S | 451.10573 | 451.10703 | 0.452 | 10.5 | 451(100.00),**83(79.94)**,136(23.65),371(22.38),356(11.46),**67(5.05)** | - | - | - | + | + | + | - |
| **D5** | methylation | 12.06 | P | C_22_H_27_O_6_ | 387.18022 | 387.18173 | 1.515 | 9.5 | 387(100.00),189(68.66),223(13.06),329(10.36),237(10.08),**69(9.11)** | - | - | - | - | - | + | - |
| **D6** | Acetylcysteine conjugation | 7.83 | N | C_26_H_30_O_9_NS | 532.16358 | 532.16437 | -0.305 | 12.5 | 403(100.00),344(12.97),373(12.12),532(11.11),329(5.22),**67(3.27)** | - | - | - | + | - | - | - |
|  |  | 7.83 | P | C_26_H_32_O_9_NS | 534.17923 | 534.17767 | -2.918 | 11.5 | 162(100.00),534(47.42),429(26.68),137(26.41),516(17.65) | - | - | - | + | + | - | - |
| **D7** | glutathionylation | 6.52 | N | C_31_H_38_O_12_N_3_S | 676.21707 | 676.21912 | 0.953 | 14.5 | 306(100.00),676(19.95),272(14.61),**67(4.49)**,388(1.58) | - | - | - | - | - | - | + |
|  |  | 6.50 | P | C_31_H_40_O_12_N_3_S | 678.23272 | 678.23248 | -0.355 | 13.5 | 308(100.00),189(21.61),219(18.87),678(15.57),371(14.35) | - | - | - | - | - | - | + |
| **E1** | oxidation and methylation | 13.17 | N | C_22_H_25_O_7_ | 401.15948 | 401.15967 | -0.906 | 10.5 | 401(100.00),**55(39.13)**,311(20.16),340(6.37),383(4.10) | - | - | - | - | + | - | - |
| **E2** | oxidation and glutathionylation | 5.66 | N | C_31_H_38_O_13_N_3_S | 692.21199 | 692.21399 | 0.908 | 14.5 | 272(100.00),**55(11.50)**,631(3.28),674(2.22),324(1.96) | - | - | - | - | - | - | + |
|  |  | 5.64 | P | C_31_H_40_O_13_N_3_S | 694.22764 | 694.22748 | -0.224 | 13.5 | 145(100.00),274(45.70),694(39.98),565(16.58),84(14.66) | - | - | - | - | - | - | + |
| **E3** | epoxidation and glutathionylation | 5.52 | N | C_31_H_40_O_13_N_3_S | 694.22764 | 694.22961 | 0.878 | 13.5 | 694(100.00),362(28.68),676(25.90),254(16.64),**55(13.22)**,388(11.10) | - | - | - | - | - | - | + |
|  |  | 5.52 | P | C_31_H_42_O_13_N_3_S | 696.24329 | 696.24176 | -2.191 | 12.5 | 151(100.00),567(38.05),696(29.54),678(29.23),137(14.00) | - | - | - | - | - | - | + |
| **E4** | hydroxylation, dehydration, and glucuronidation | 7.29 | N | C_27_H_29_O_12_ | 545.16535 | 545.16492 | -2.806 | 13.5 | 387(100.00),**55(51.22)**,369(40.11),372(21.81),137(16.67),545(3.55) | - | - | - | - | - | - | + |
| **E5** | dihydroxylation and glucuronidation | 5.86 | N | C_27_H_31_O_14_ | 579.17083 | 579.17279 | 1.487 | 12.5 | 271(100.00),579(27.92),295(2.89),152(2.77),**55(1.31)**,314(0.83) | - | - | - | - | - | - | + |
| **F1** | demethylation and  glucuronidation | 7.15 | N | C_26_H_29_O_12_ | 533.16535 | 533.16724 | 1.483 | 12.5 | 533(100.00),113(45.12),**39(42.33)**,357(36.63) | + | + | + | + | - | - | - |
| **F2** | demethylation and sulfation | 7.69 | N | C_20_H_21_O_9_S | 437.09008 | 437.09174 | 1.290 | 10.5 | 437(100.00),357(57.45),**39(49.63)**,136(13.69),342(5.72) | + | - | + | + | + | - | - |
| **F3** | demethylation, hydroxylation, and  glucuronidation | 5.60 | N | C_26_H_29_O_13_ | 549.16027 | 549.16223 | 1.577 | 12.5 | 549(100.00),113(31.26),**39(26.15)**,329(16.77),373(5.15) | - | + | - | - | - | - | - |
| **F4** | demethylation,  hydroxylation, and sulfation | 6.52 | N | C_20_H_21_O_10_S | 453.08499 | 453.08624 | 0.149 | 10.5 | 453(100.00),373(54.59),**39(27.55)**,358(20.68),137(15.05) | - | - | - | + | + | - | - |
| **F5** | demethylation and glycinization | 8.21 | N | C_22_H_24_O_7_N | 414.15473 | 414.15884 | 3.015 | 11.5 | 162(100.00),207(59.69),414(52.96),285(34.53),**39(24.92)** | - | - | - | + | - | - | - |
| **F6** | demethylation and glutathionylation | 5.61 | N | C_30_H_36_O_12_N_3_S | 662.20142 | 662.20325 | 1.106 | 14.5 | 306(100.00),272(9.58),662(8.33),254(7.64),**39(6.38)** | - | - | - | - | - | - | + |
|  |  | 5.60 | P | C_30_H_38_O_12_N_3_S | 664.21707 | 664.21606 | -1.011 | 13.5 | 137(100.00),308(95.13),179(42.74),664(42.37) | - | - | - | - | - | - | + |
| **F7** | demethylation and  glutathionylation | 6.10 | N | C_30_H_36_O_12_N_3_S | 662.20142 | 662.20306 | 0.819 | 14.5 | 662(100.00),272(25.41),254(11.93),306(9.93) | - | - | - | - | - | - | + |
|  |  | 6.10 | P | C_30_H_38_O_12_N_3_S | 664.21707 | 664.21552 | -1.551 | 13.5 | 535(100.00),151(85.86),664(67.87),589(27.77),**41(22.84)** | - | - | - | - | - | - | + |
| **F8** | demethylation and  glutathionylation | 6.30 | N | C_30_H_36_O_12_N_3_S | 662.20142 | 662.20337 | 1.288 | 14.5 | 389(100.00),272(45.57),153(20.32),662(8.70),**39(4.50)** | - | - | - | - | - | - | + |
|  |  | 6.29 | P | C_30_H_38_O_12_N_3_S | 664.21707 | 664.21613 | -0.941 | 13.5 | 535(100.00),151(99.08),664(76.44),589(25.43),**41(14.36)** | - | - | - | - | - | - | + |
| **F9** | demethylation, oxidation and  glutathionylation | 5.40 | N | C_30_H_36_O_13_N_3_S | 678.19634 | 678.19513 | -2.302 | 14.5 | 271(100.00),678(10.83),254(10.49),373(10.07),359(8.08),137(3.27) | - | - | - | - | - | - | + |
|  |  | 5.39 | P | C_30_H_38_O_13_N_3_S | 680.21199 | 680.21057 | -1.415 | 13.5 | 535(100.00),664(76.44),589(25.43),**41(14.36)** | - | - | - | - | - | - | + |
| **F10** | demethylation, oxidation and  glutathionylation | 5.76 | N | C_30_H_36_O_13_N_3_S | 678.19634 | 678.19727 | -0.162 | 14.5 | 272(100.00),143(48.17),405(32.45),**39(27.80)**,218(21.92),387(12.07) | - | - | - | - | - | - | + |
|  |  | 5.76 | P | C_30_H_38_O_13_N_3_S | 680.21199 | 680.21094 | -1.045 | 13.5 | 145(100.00),680(44.23),274(25.37),551(15.11),**41(9.70)** | - | - | - | - | - | - | + |
| **F11** | demethylation, oxidation and  glutathionylation | 5.58 | N | C_30_H_36_O_13_N_3_S | 678.19634 | 678.19830 | 0.868 | 14.5 | 272(100.00),405(15.44),678(15.31)387(3.95),373(1.21),**39(1.18)** | - | - | - | - | - | - | + |
|  |  | 5.57 | P | C_30_H_38_O_13_N_3_S | 680.21199 | 680.21075 | -1.235 | 13.5 | 145(100.00),680(34.87),274(33.80),551(24.96),137(13.14),**41(9.88)** | - | - | - | - | - | - | + |
| **F12** | demethylation and diglucuronidation | 7.64 | N | C_32_H_37_O_18_ | 709.19744 | 709.19794 | -0.842 | 14.5 | 345(76.84),388(71.94),373(45.54),**39(7.80)**,709(5.55) | - | - | - | - | - | - | + |
|  |  | 7.63 | P | C_32_H_39_O_18_ | 711.21309 | 711.21289 | -0.282 | 13.5 | 405(100.00),375(52.70),347(6.06),711(5.96) | - | - | - | - | - | - | + |
| **F13** | didemethylation and glucuronidation | 5.76 | N | C_25_H_27_O_12_ | 519.14970 | 519.15094 | 0.271 | 12.5 | 519(100.00),**39(42.32)**,343(39.41),175(19.39),137(4.29) | - | - | - | + | - | - | - |
| **F14** | didemethylation and glucuronidation | 5.94 | N | C_25_H_27_O_12_ | 519.14970 | 519.15082 | 0.040 | 12.5 | 519(100.00),343(39.85),**39(35.89)**,313(1.56),297(0.95) | - | - | - | + | - | - | - |
| **F15** | didemethylation and glucuronidation | 6.12 | N | C_25_H_27_O_12_ | 519.14970 | 519.15106 | 0.502 | 12.5 | 519(100.00),343(51.51),**39(17.15)**,393(15.11),297(2.27) | - | - | - | + | - | - | - |
| **F16** | didemethylation and glucuronidation | 6.37 | N | C_25_H_27_O_12_ | 519.14970 | 519.15100 | 0.386 | 12.5 | 519(100.00),343(49.89),**39(27.86)**,313(8.59),297(4.27),393(1.85) | - | - | - | + | - | - | - |
| **F17** | didemethylation and glucuronidation | 6.55 | N | C_25_H_27_O_12_ | 519.14970 | 519.15100 | 0.386 | 12.5 | 519(100.00),343(58.37),**39(30.28)**,313(6.05),297(2.27),393(1.89) | - | - | - | + | - | - | - |
| **F18** | didemethylation, dehydroxylation, and glucuronidation | 6.62 | N | C_25_H_27_O_11_ | 503.15479 | 503.15683 | 1.879 | 12.5 | 503(100.00),**39(44.54)**,175(29.77),327(27.45)283(7.49) | + | - | - | + | - | + | - |
| **F19** | didemethylation, dehydroxylation, demethoxy,and glucuronidation | 6.82 | N | C_24_H_25_O_10_ | 473.14422 | 473.14606 | 1.564 | 12.5 | 473(100.00),297(55.37),**39(35.96)**,253(16.87),121(5.45) | + | + | + | + | - | + | - |
| **F20** | didemethylation, dehydroxylation, demethoxy,and glucuronidation | 7.00 | N | C_24_H_25_O_10_ | 473.14422 | 473.14563 | 0.655 | 12.5 | 473(100.00),297(45.40),**39(33.47)**,253(14.43),219(1.94) | + | + | + | + | - | + | - |
| **F21** | tri-demethylation and glucuronidation | 5.53 | N | C_24_H_25_O_12_ | 505.13405 | 505.13528 | 0.259 | 12.5 | 329(100.00),505(90.35),113(26.04),207(22.66),**39(20.76)** | - | - | - | + | - | - | - |
| **F22** | tri-demethylation and diglucuronidation | 6.27 | N | C_30_H_33_O_18_ | 681.16614 | 681.16809 | 1.252 | 14.5 | 360(100.00),537(64.55),681(51.56),**39(45.50)**,345(43.92),619(12.86) | - | - | - | - | - | - | + |
| **F23** | tri-demethylation, dehydroxylation, and glucuronidation | 6.47 | N | C_24_H_25_O_11_ | 489.13914 | 489.14130 | 2.178 | 12.5 | 489(100.00),313(90.34),**39(24.08)**,201(21.82),269(3.29),252(2.82) | + | + | + | + | - | - | - |
| **F24** | tri-demethylation, dehydroxylation, and diglucuronidation | 5.36 | N | C_30_H_33_O_17_ | 665.17123 | 665.17242 | 0.146 | 14.5 | 489(100.00),313(92.08),665(37.80),**39(29.04)**,269(13.91) | - | - | - | + | - | - | - |
| **F25** | Hydrogenation and glucuronidation | 2.28 | N | C_27_H_35_O_12_ | 551.21230 | 551.21313 | -0.489 | 10.5 | 119(100.00),220(63.60),551(11.54),**39(7.98)** | - | - | - | - | + | - | - |
| **F26** | tri-demethoxy and glucuronidation | 7.44 | N | C_24_H_25_O_9_ | 457.14931 | 457.14960 | -1.762 | 12.5 | 389(100.00),**39(63.16)**,457(23.02),331(12.98),345(8.56),371(6.85) | - | + | + | - | - | - | - |
| **F27** | internal hydrolysis and glucuronidation | 5.93 | N | C_27_H_33_O_13_ | 565.19342 | 565.19157 | 1.337 | 11.5 | 565(100.00),**39(32.42)**,330(29.41),253(19.34),136(7.30) | + | + | + | - | - | - | - |
| **F28** | internal hydrolysis and sulfation | 6.31 | N | C_21_H_25_O_10_S | 469.11629 | 469.11743 | 0.083 | 9.5 | 469(100.00),389(14.30),**39(8.04)**,423(7.41),254(6.90),136(6.54) | - | - | - | - | + | - | - |
| **F29** | internal hydrolysis and cysteinization | 6.17 | P | C_24_H_32_O_8_NS | 494.18431 | 494.18298 | -0.028 | 9.5 | 151(100.00),373(42.88),476(39.75),494(28.01),458(11.94),**41(5.80)** | - | - | - | + | + | - | - |
| **F30** | internal hydrolysis, decarbonylation, and glucuronidation | 4.45 | N | C_26_H_33_O_12_ | 537.19665 | 537.19891 | 2.160 | 10.5 | 119(100.00),361(59.74),**39(12.58)**,537(10.31) | - | - | - | - | + | - | - |
| **F31** | internal hydrolysis,  oxidation, and glutathionylation | 4.79 | N | C_31_H_40_O_14_N_3_S | 710.22255 | 710.22491 | 1.779 | 13.5 | 710(78.25),403(11.22),692(10.03),360(5.12),646(2.70),345(2.36),**39(1.07)** | - | - | - | - | - | - | + |
| **F32** | internal hydrolysis,  oxidation, and glutathionylation | 5.02 | N | C_31_H_40_O_14_N_3_S | 710.22255 | 710.22443 | 1.103 | 13.5 | 272(100.00),143(74.12),692(54.15),710(49.86),464(18.48),**39(12.57)** | - | - | - | - | - | - | + |
|  |  | 5.00 | P | C_31_H_42_O_14_N_3_S | 712.23820 | 712.23853 | 0.463 | 12.5 | 145(100.00),274(58.89),712(32.42),181(18.13),**41(14.02)** | - | - | - | - | - | - | + |
| **F33** | internal hydrolysis,  dehydrogenation,and glutathionylation | 5.66 | N | C_31_H_38_O_13_N_3_S | 692.21199 | 692.21399 | 1.312 | 14.5 | 272(100.00),385(55.60),692(49.71),**39(11.50)**,631(3.28),674(2.22) | - | - | - | - | - | - | + |
|  |  | 5.64 | P | C_31_H_40_O_13_N_3_S | 694.22764 | 694.22705 | -0.843 | 13.5 | 145(100.00),274(45.70),694(32.63),136(11.87),**41(8.52)** | - | - | - | - | - | - | + |

Note: t_R_: retention time; PS: SPE column treated plasma; PM: Methanol treated plasma; PA: Acetonitrile treated plasma; U: urine; F: faeces; L: liver; LM: liver microsomes; “+”: detected; “-”: undetected
